# Inorganic Carbon Modulates Emulsification Activity and Transcriptional Responses in *Vreelandella zhaodongensis* BS253

**DOI:** 10.3390/molecules31122182

**Published:** 2026-06-22

**Authors:** Amanda Pasinato Napp, Henrique Alves de Brito, Daniel Ubiratan Haas de Brito, Eduarda Vargas Abati, Francine Melise dos Santos, Clarissa Lovato Melo, João Pedro Tauscheck Zielinski, Charley Christian Staats

**Affiliations:** 1 Institute of Petroleum and Natural Resources (IPR), Pontifical Catholic University of Rio Grande do Sul (PUCRS), Porto Alegre 90619-900, Rio Grande do Sul, Brazil; amanda.pasinato@pucrs.br (A.P.N.); henrique.brito@pucrs.br (H.A.d.B.); daniel.ubiratan@pucrs.br (D.U.H.d.B.); eduarda.abati@pucrs.br (E.V.A.);; 2 Graduate Program in Cellular and Molecular Biology, Center of Biotechnology, Federal University of Rio Grande do Sul, Porto Alegre 91501-970, Rio Grande do Sul, Brazil

**Keywords:** haloalkaliphilic bacteria, *Vreelandella*, inorganic carbon, emulsification, transcriptome

## Abstract

Inorganic carbon availability is an underexplored factor influencing extracellular emulsification-associated responses in haloalkaliphilic bacteria. Here, we show that *Vreelandella zhaodongensis* BS253 exhibits distinct physiological and transcriptional responses to CO_2_ enrichment and bicarbonate supplementation, accompanied by condition-dependent changes in emulsification activity. Both moderate CO_2_ enrichment (5–10%) and NaHCO_3_ supported high emulsification values (E24 > 60%). However, CO_2_ favored higher emulsification activity relative to biomass, whereas NaHCO_3_ promoted greater biomass accumulation and elevated absolute activity. Transcriptomic profiling revealed extensive condition-dependent reprogramming, particularly involving membrane transport, envelope-associated functions, and genes annotated as related to exopolysaccharide biosynthesis. Integrative phenotype-guided analyses prioritized candidate genes statistically associated with the emulsification phenotype. The extracellular emulsification-active material remained active across a broad range of salinity, temperature, pH, and pressure, demonstrating pronounced physicochemical robustness. Together, these findings indicate that inorganic carbon availability modulates emulsification activity and associated transcriptional responses in a haloalkaliphile and highlight extremophilic bacteria as promising platforms for sustainable bioprocesses based on inorganic carbon inputs.

## 1. Introduction

Extremophilic microorganisms inhabiting saline and alkaline environments have emerged as promising platforms for robust biotechnological processes. Members of the family Halomonadaceae are particularly attractive due to their metabolic versatility, tolerance to high salinity and alkaline conditions, and capacity to produce industrially relevant biomolecules, including exopolysaccharides, compatible solutes, and surface-active compounds [1,2,3]. Within this family, the genus *Vreelandella* is generally described as Gram-negative rods with aerobic or facultatively anaerobic metabolism and halophilic, alkaliphilic, or alkali-tolerant traits [2]. These physiological features make *Vreelandella* a relevant candidate for bioprocesses operating under saline, alkaline, or otherwise harsh conditions, where conventional microorganisms may be less competitive or stable.

Microbial surface-active compounds play key roles in stress adaptation and interfacial processes and support sustainable, bio-based production systems [4,5]. Compared with synthetic surfactants, these compounds offer several advantages, such as biodegradability, low toxicity, and the possibility of production from renewable resources [6]. However, microbial surface-active compounds comprise chemically and functionally diverse molecules. Low-molecular-weight biosurfactants typically reduce surface and interfacial tension, whereas high-molecular-weight bioemulsifiers mainly stabilize emulsions between immiscible phases and may have little or no effect on surface tension. Among microbial surface-active molecules, bioemulsifiers are especially interesting because of their ability to maintain stable emulsions under challenging environmental conditions [7,8,9]. These properties make them attractive for applications in bioremediation, enhanced oil recovery, food processing, and pharmaceutical formulations [10,11].

Recent studies have also highlighted the potential of extremophilic microorganisms in processes related to carbon capture, utilization, and storage (CCUS). Inorganic carbon sources such as CO_2_ and bicarbonate can influence microbial metabolism and modulate extracellular emulsification-associated phenotypes [12,13,14,15]. However, the molecular and physiological mechanisms linking inorganic carbon availability to emulsification activity remain poorly understood, particularly in haloalkaliphilic bacteria. This knowledge gap limits both our understanding of microbial adaptation to carbon-rich environments and the rational development of sustainable bioprocesses based on inorganic carbon inputs.

The Brazilian Pantanal contains numerous saline–alkaline lakes characterized by high evaporation rates, limited water exchange, and strong seasonal fluctuations. These conditions contribute to the selection of specialized microbial communities capable of tolerating high salinity, alkaline pH, and fluctuating oxygen and nutrient availability [16,17]. As a result, these environments represent an underexplored reservoir of microbial diversity with significant biotechnological potential [18].

In this study, we investigated the effect of inorganic carbon availability on emulsification activity in the haloalkaliphilic bacterium *Vreelandella zhaodongensis* BS253, isolated from a hypersaline lake in the Brazilian Pantanal. We combined phenotypic assays with transcriptomic analysis to identify physiological and transcriptional responses associated with CO_2_-enriched conditions and bicarbonate supplementation. In addition, the physicochemical stability of the extracellular emulsification-active material was evaluated to assess its functional robustness under stress conditions. We hypothesized that inorganic carbon availability modulates the emulsification phenotype through coordinated physiological and transcriptional responses involving extracellular matrix-associated functions, membrane transport, and carbon-associated cellular adaptation. These findings provide new insights into inorganic carbon-associated emulsification responses and highlight extremophilic bacteria as promising platforms for sustainable bioprocesses.

## 2. Results

### 2.1. Strain BS253 Tolerates Oxygen Limitation and Inorganic Carbon-Enriched Conditions

Strain BS253 was able to grow under anaerobic conditions, forming visible colonies after 21 days of incubation. Growth was also observed on MSM plates supplemented with NaHCO_3_ and under a CO_2_-enriched atmosphere (Figure S1). These observations indicated metabolic flexibility under anaerobic and inorganic carbon conditions, prompting further screening of emulsification activity under different inorganic carbon sources.

### 2.2. Cultivation Conditions Affect Emulsification Activity Under Inorganic Carbon Enrichment

**Figure 1:**
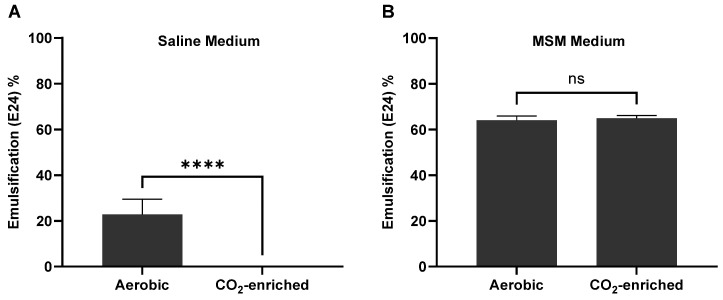
Emulsification activity by strain BS253 under different conditions: (**A**) Emulsification activity in the original saline medium used for strain isolation under aerobic conditions and a 5% CO_2_-enriched atmosphere after 24 h of incubation. (**B**) Emulsification activity in MSM broth under NaHCO_3_-supplemented aerobic conditions and 5% CO_2_-enriched atmosphere after 72 h of incubation. Cultures were incubated at 28 °C at 180 rpm. Data are presented as mean ± standard deviation from three independent experiments. Statistical significance was assessed using Student’s *t*-test (panel **A**) and Mann–Whitney test (panel **B**); **** *p* < 0.0001 and ns, not significant.

Emulsification activity (E24) by strain BS253 was first evaluated in the original saline medium used for strain isolation. Under this condition, E24 was detected only under aerobic cultivation, reaching 22.8%, whereas no detectable activity was observed under a 5% CO_2_-enriched atmosphere (Figure 1A). In contrast, cultivation of strain BS253 in MSM resulted in E24 values above 60% under both aerobic conditions supplemented with NaHCO_3_ and a 5% CO_2_-enriched atmosphere, with no significant differences between the two conditions (Figure 1B). These results show that emulsification activity varied according to the cultivation medium and inorganic carbon conditions tested. Although emulsification activity was detected under selected cultivation conditions, no substantial reduction in surface tension was observed in strain BS253 cultures.

**Figure 2:**
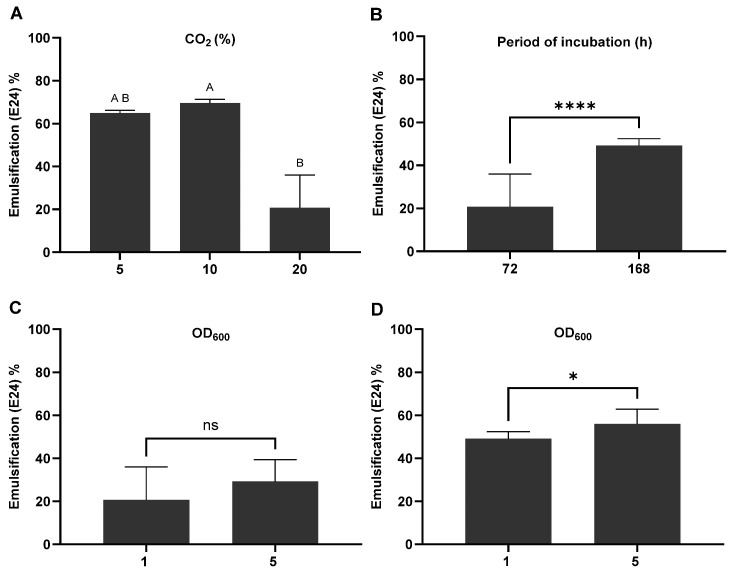
Emulsification activity of strain BS253 under different CO_2_-enrichment concentrations: (**A**) Effect of CO_2_ concentration (5, 10, and 20%) on emulsification activity after 3 days of cultivation in MSM medium using an inoculum adjusted to OD_600_ = 1. (**B**) Comparison of emulsification activity under 20% CO_2_ after 3 and 6 days of cultivation. (**C**) Effect of inoculum density (OD_600_ = 1 and 5) on emulsification activity after 3 days under 20% CO_2_. (**D**) Effect of inoculum density (OD_600_ = 1 and 5) on emulsification activity after 6 days under 20% CO_2_. Cultures were incubated in MSM medium at 28 °C and 180 rpm. Data are presented as mean ± standard deviation from three independent experiments. Statistical significance was assessed using the Kruskal–Wallis test (panel **A**), Student’s *t*-test (panels **B**,**C**), and Mann–Whitney test (panel **D**). In (**A**), bars sharing the same letters indicate no statistically significant differences among groups. In (**B**,**D**), * represents *p* < 0.05, and **** represents *p* < 0.0001.

Following the identification of high emulsification activity under a 5% CO_2_-enriched atmosphere, the emulsification profile of strain BS253 was further evaluated under increasing CO_2_ concentrations. Emulsification activity remained above 60% under 5% and 10% CO_2_, with no significant difference among the tested conditions. In contrast, 20% CO_2_ markedly reduced emulsification activity, which reached 20.7% after 72 h of incubation (Figure 2A). Extension of the cultivation period from 72 to 168 h under 20% CO_2_ resulted in a partial recovery of emulsification activity, increasing to 49.1% (Figure 2B). Similarly, increasing the initial cell density from OD_600_ 1 to 5 under 20% CO_2_ also led to higher activity levels, reaching 55.9% after 168 h (Figure 2C,D).

### 2.3. The Extracellular Emulsification-Active Material Remains Active Under a Broad Range of Physicochemical Conditions

**Figure 3:**
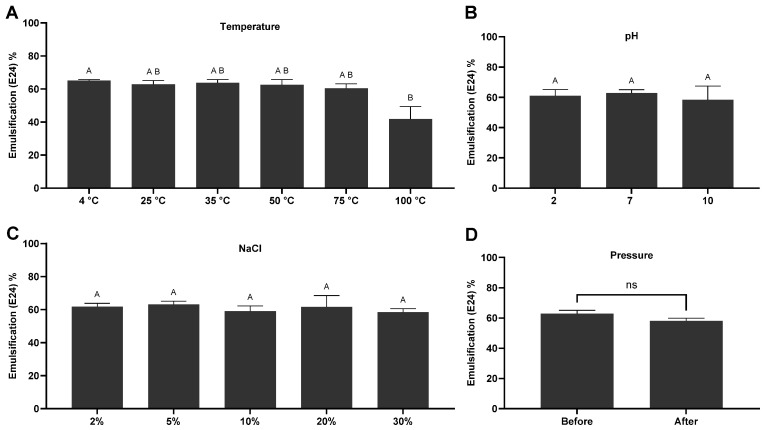
Physicochemical stability of the extracellular emulsification-active material obtained from *Vreelandella zhaodongensis* BS253 under different environmental conditions: (**A**) Thermal stability was evaluated at temperatures ranging from 4 °C to 100 °C. (**B**) Stability across different pH conditions (pH 2, 7, and 10). (**C**) Stability under different NaCl concentrations (2–30). (**D**) Stability after thermal treatment and pressure conditions (121 °C and 2 atm for 15 min). Cultures were incubated in MSM medium at 28 °C and 180 rpm. Data are presented as mean ± standard deviation from three independent experiments. Statistical significance was assessed using the Kruskal–Wallis test for panels (**A**,**B**), ordinary one-way ANOVA for panel (**C**), and Student’s *t*-test for panel (**D**). Bars sharing the same letters indicate no statistically significant differences among groups.

For these stability assays, E24 was measured using a solubilized extracellular preparation filtered through 0.22 µm membranes before exposure to the different physicochemical treatments. The extracellular emulsification-active material from strain BS253 showed high stability over a wide temperature range, with emulsification activity remaining above 60% after incubation at 4, 25, 37, 50, and 75 °C. At 100 °C, E24 decreased to 46% and was significantly lower than the activity observed at 4 °C, although intermediate temperatures showed overlapping statistical groups (Figure 3A). These results indicate that the extracellular preparation retained substantial emulsification activity up to 75 °C and partially retained activity after exposure to 100 °C. Emulsification activity was maintained across a wide pH range, with the highest activity showing no significant difference (Figure 3B). Similarly, emulsification activity was maintained across all salinity conditions tested, from 2% to 30% NaCl, with values remaining above 50% (Figure 3C). Notably, exposure to combined high temperature and pressure (121 °C, 2 atm) did not markedly affect emulsifying activity, with E24 remaining at 58% (Figure 3D).

### 2.4. Carbon Availability Modulates Growth and Emulsification Activity over Time

**Figure 4:**
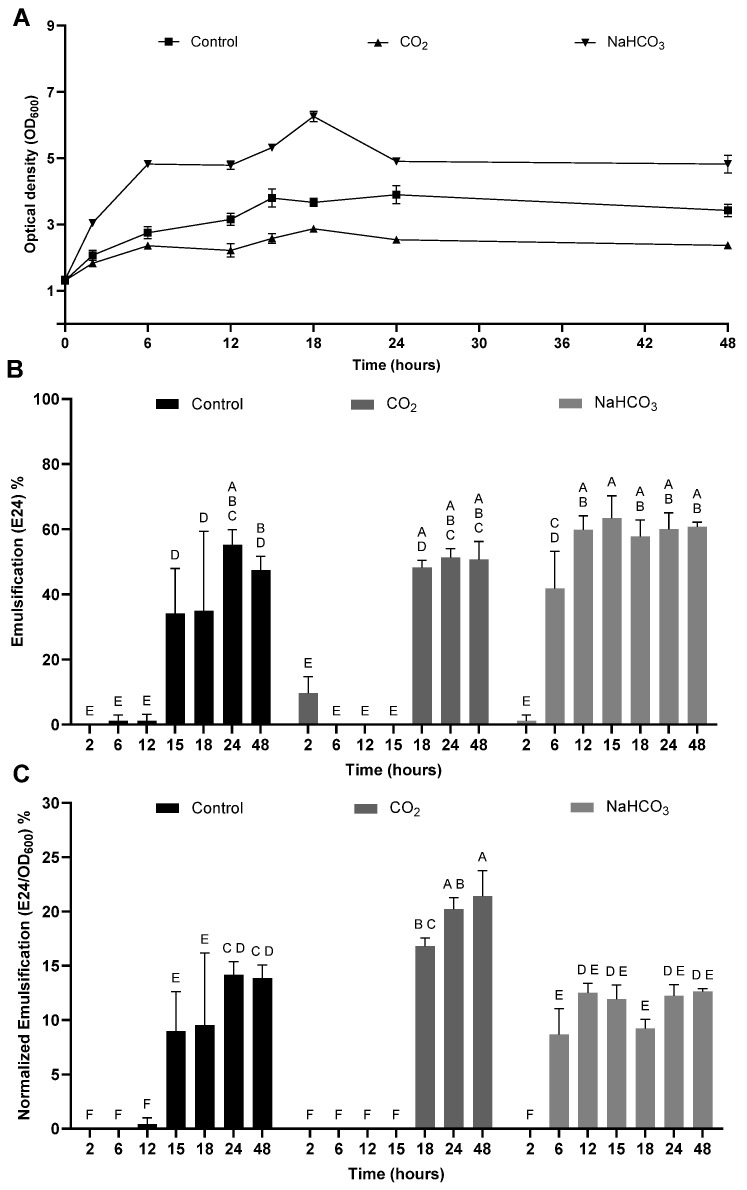
Time-course analysis of microbial growth and emulsification activity in *Vreelandella zhaodongensis* BS253 under different inorganic carbon conditions: (**A**) Microbial growth curves expressed as optical density at 600 nm. (**B**) Emulsification (E24) was measured over time under different cultivation conditions. (**C**) Normalized emulsification (E24/OD_600_), representing the specific emulsification activity relative to cell density. Cultures were incubated in MSM broth at 28 °C and 180 rpm. Data are presented as mean ± standard deviation from three independent experiments. Statistical significance was assessed using ordinary one-way ANOVA for panel (**A**) and two-way ANOVA for panels (**B**,**C**). Bars sharing the same letters indicate no statistically significant differences among groups.

Time-course experiments showed that both microbial growth and emulsification activity varied according to the inorganic carbon condition (Figure 4A,B). In MSM under aerobic conditions without additional inorganic carbon supplementation (control), OD_600_ increased from 2.0 to 3.8 within the first 15 h and remained constant thereafter. Under this condition, emulsification activity was negligible during the early stages of cultivation, increased after 15 h, and reached 55.2% at 24 h, remaining constant until 48 h. Cultures incubated under 10% CO_2_ exhibited the lowest biomass accumulation with OD_600_ values ranging from 1.8 to 2.9 throughout the experiment. Emulsification activity under these conditions increased only at later cultivation stages, reaching 48.3% at 18 h and remaining elevated thereafter. In contrast, cultures grown in MSM supplemented with 2% NaHCO_3_ showed the highest biomass accumulation, with OD_600_ reaching 6.3. Under this condition, emulsification activity was detected earlier, reaching 41.8% at 6 h and peaking at 63.4% at 15 h, with values remaining close to 60% until the end of the experiment.

To compare emulsification activity relative to biomass accumulation, E24 values were normalized by OD_600_ (Figure 4C). Interestingly, cultures grown under 10% CO_2_ showed the highest normalized values, exceeding 20%/OD between 18 h and 48 h. In the control condition, normalized values reached approximately 14%/OD during the same period. In contrast, NaHCO_3_-supplemented cultures showed normalized values of approximately 12%/OD. Based on these trajectories, 12 h and 18 h were selected as physiologically informative stages for transcriptomic profiling.

### 2.5. Global Transcriptomic Response Varies According to Cultivation Time and Inorganic Carbon Conditions

**Figure 5:**
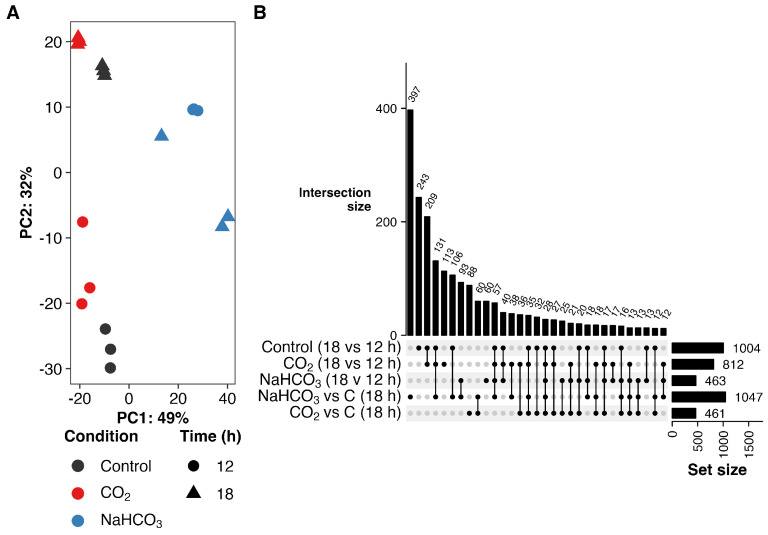
Global transcriptomic responses under different cultivation conditions: (**A**) Principal component analysis (PCA) of global gene expression profiles showing sample clustering according to cultivation conditions and sampling time. Colors indicate cultivation conditions, and symbols represent sampling time. (**B**) UpSet plot showing the intersections among differentially expressed genes (DEGs) identified in the comparisons Control (18 vs. 12 h), CO_2_ (18 vs. 12 h), NaHCO_3_ (18 vs. 12 h), NaHCO_3_ vs. Control (18 h), and CO_2_ vs. Control (18 h). Vertical bars indicate the number of shared DEGs for each intersection, connected dots indicate the comparisons included in each intersection, and horizontal bars represent the total number of DEGs in each comparison. DEGs were defined as genes with |log_2_ fold change| > 1 and padj < 0.05.

To characterize the molecular events associated with emulsification activity under inorganic carbon conditions, RNA-seq analysis was performed using samples collected at 12 h and 18 h from cultures grown in MSM (Control), MSM supplemented with NaHCO_3_ (NaHCO_3_), and MSM under a CO_2_-enriched atmosphere (CO_2_). Principal component analysis (PCA) of normalized gene expression data showed a clear separation of samples according to both cultivation time and carbon source (Figure 5A). Hierarchical clustering based on the 100 most variable genes showed a similar organization of samples, with samples grouping by condition and time point and consistent clustering of biological replicates (Figure S2).

To evaluate the overlap and specificity of transcriptional responses, gene set intersections across five experimental comparisons were analyzed (Figure 5B). The number of differentially expressed genes (DEGs) varied among comparisons, with the largest sets observed in NaHCO_3_ vs. Control (18 h) (1047 genes) and Control (18 vs. 12 h) (1004 genes). Smaller DEG sets were identified in CO_2_ vs. Control (18 h) (461 genes) and NaHCO_3_ (18 vs. 12 h) (463 genes).

Intersection analysis identified a core set of 397 genes shared across all five experimental conditions, representing a common set of genes across all conditions. Furthermore, a distinct subset of 40 genes was shared exclusively among the three temporal comparisons (Control, CO_2_, and NaHCO_3_ at 18 vs. 12 h), likely representing a general time-dependent response independent of the specific treatment. Similarly, a total of 60 genes were shared between the two 18 h treatment-versus-control comparisons (NaHCO_3_ vs. Control and CO_2_ vs. Control), highlighting a common response to carbon-source or pH modulation at that time point. These results showed that a substantial portion of the transcriptome exhibited similar responses across all conditions, whereas the NaHCO_3_ treatment at 18 h was associated with the largest and most distinct set of transcriptional changes.

To further explore pathway-level responses beyond the differentially expressed gene sets, carbon metabolism-related genes were examined due to their potential effect on growth activity in CO_2_ and NaHCO_3_ conditions. Although these genes were not identified as differentially expressed in the evaluated contrasts, their expression varied across conditions and time points, including a slightly reduced carbonic anhydrase expression at 18 h under NaHCO_3_ conditions relative to 12 h (Figure S3).

**Figure 6:**
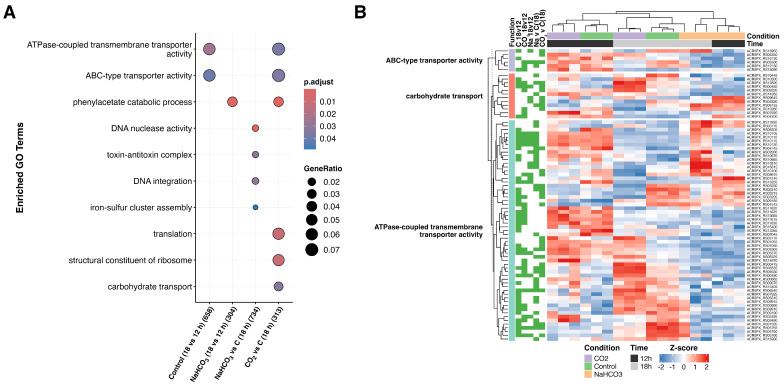
Functional enrichment and expression patterns of selected differentially expressed genes under distinct cultivation conditions: (**A**) Bubble plot showing enriched Gene Ontology (GO) terms associated with differentially expressed genes (DEGs) identified in the comparisons of Control (18 h vs. 12 h), NaHCO_3_ (18 h vs. 12 h), NaHCO_3_ vs. Control (18 h), and CO_2_ vs. Control (18 h). The Y-axis lists the enriched GO terms, and the X-axis indicates experimental comparisons. Bubble size represents the gene ratio, and bubble color indicates the adjusted *p*-value, with warmer colors corresponding to higher statistical significance. (**B**) Heatmap showing the expression profiles of genes associated with selected enriched functions across all samples. Rows represent genes, and columns represent samples grouped by cultivation condition and sampling time. The dendrograms indicate hierarchical clustering of genes and samples based on expression similarity. The side annotation marks the functional category assigned to each gene, and the top annotations indicate condition and time. Colors represent row-scaled expression values (Z-score), ranging from lower expression (blue) to higher expression (red).

Therefore, Gene Ontology (GO) enrichment analysis was performed to identify biological processes associated with the observed transcriptional changes. In the comparison between NaHCO_3_-supplemented medium and basal medium at 18 h, enriched GO terms included DNA nuclease activity and toxin–antitoxin complex. GO terms related to ABC-type transporter activity and ATPase-coupled transmembrane transporter activity were enriched both in the 18 h vs. 12 h comparison in basal medium and in the CO_2_ vs. basal medium comparison at 18 h (Figure 6A). In addition, genes associated with the enriched transporter-related functions displayed distinct expression patterns across cultivation conditions and sampling times (Figure 6B). Hierarchical clustering revealed that several genes related to ABC-type transporter activity, ATPase-coupled transmembrane transporter activity, and carbohydrate transport grouped into coherent expression modules, showing coordinated regulation. In particular, transporter-associated genes generally exhibited higher expression under specific condition–time combinations.

In summary, global transcriptomic analysis showed a clear separation of gene expression according to cultivation time and carbon availability, with NaHCO_3_ associated with the most extensive and distinct transcriptional profile. A core set of genes exhibited consistent expression across all conditions, while transporter-related genes displayed condition- and time-dependent enrichment and clustering, particularly at 18 h.

### 2.6. Genes Annotated as EPS-Related Exhibit Distinct Expression Patterns

The high emulsification stability, combined with the lack of substantial surface tension reduction, is compatible with the presence of high-molecular-weight bioemulsifier-like material, including extracellular polysaccharides (EPSs), as reported for some *Halomonas* species [19,20], close phylogenetic relatives of *Vreelandella* species [2].

However, because EPS quantification and compositional analyses were not performed, the EPS-related nature of the active material remains a working hypothesis. Preliminary qualitative biochemical screening was performed using purified and lyophilized extracellular fractions. The assays indicated carbohydrate-associated and amino-containing components, as well as anionic functional groups, while neutral lipid-associated signals were limited (Figure S5). To explore whether the emulsification phenotype could be associated with extracellular polymer-related functions, we screened the genome annotation for genes putatively involved in EPS precursor synthesis, assembly, and export. The search prioritized genes categorized under the COG category M (Cell wall/membrane/envelope biogenesis) and those containing keywords related to polysaccharide assembly, including glycosyltransferases, flippases, polymerases, and sugar-nucleotide metabolism (e.g., *wzx*, *wzy*, and *wza*). The rationale for this targeted analysis was to evaluate whether the increased emulsification activity observed under inorganic carbon enrichment was accompanied by transcriptional modulation of genes annotated as related to extracellular polymer biosynthesis. The genomic screening revealed a repertoire of genes annotated as potentially related to EPS production (Table S1), suggesting the presence of both Wzy-dependent and ABC transporter-dependent assembly mechanisms. Key identified components included several glycosyltransferases (GTs), which may participate in the assembly of polysaccharide repeating units, as well as proteins annotated as related to export machinery. Furthermore, the identification of genes involved in activated sugar-nucleotide precursor metabolism indicates that BS253 possesses genomic components compatible with extracellular polymer biosynthesis. However, these data do not demonstrate direct conversion of inorganic carbon into complex extracellular polymers, which would require targeted biochemical or isotope-labeling analyses.

Transcriptomic profiling of the selected genes (Figure S4) demonstrated that these EPS-related targets displayed condition-specific expression patterns. While many genes showed stable or declining expression in the control medium over time, cultivation under NaHCO_3_ and CO_2_-enriched conditions was associated with transcriptional modulation at 18 h. Hierarchical clustering identified a module of EPS-related genes, including several glycosyltransferases and export proteins, that showed increased expression under inorganic carbon-enriched conditions. Notably, the NaHCO_3_ vs. Control (18 h) comparison showed the largest set of differentially expressed genes (DEGs) among the conditions analyzed.

### 2.7. Integrative Analysis Prioritizes Candidate Genes Associated with Emulsification Activity

To prioritize genes statistically associated with the emulsification phenotype, a multi-layered framework integrating statistical evidence, phenotype association, and machine learning-based predictive modeling was implemented. This approach focused on the set of differentially expressed genes (DEGs) identified across all experimental contrasts and evaluated each gene using three complementary criteria: (i) evidence counts, representing the number of contrasts in which a gene was significantly differentially expressed; (ii) adjusted Pearson correlation (r) between transcript abundance and the emulsification index, calculated after removing the effects of experimental condition and time; and (iii) Random Forest (RF) regression, which quantified the predictive importance of each gene based on its contribution to model accuracy (%IncMSE). To minimize confounding effects associated with experimental design, both gene expression profiles and phenotypic values were subjected to residualization prior to correlation and machine learning analyses. This adjustment resulted in a slightly reduced but more biologically meaningful correlation range (*r* = −0.833 to 0.806), with 823 genes still exhibiting strong associations (|r| > 0.5). These results indicate that a substantial portion of the transcriptome remains statistically associated with the emulsification phenotype independent of condition- and time-specific effects.

**Figure 7:**
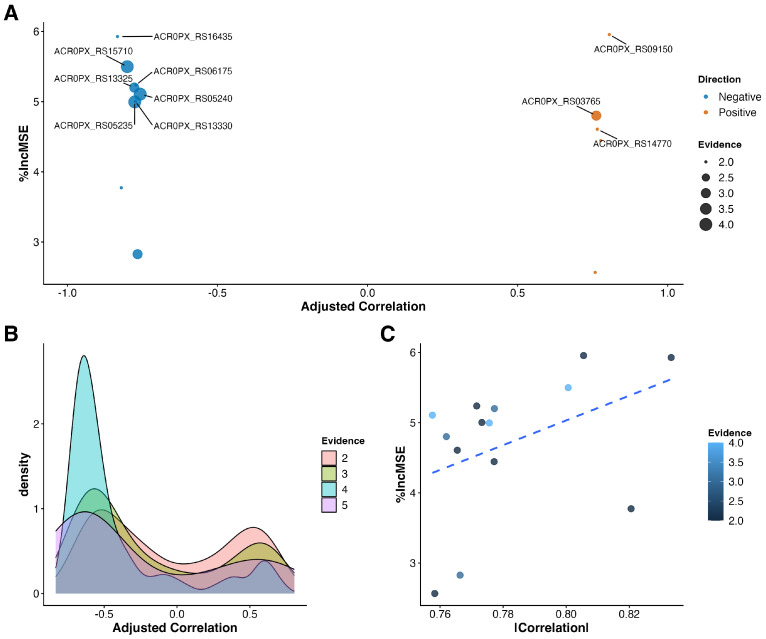
Integrative prioritization of genes associated with emulsification: (**A**) Multi-dimensional scatter plot showing the relationship between adjusted Pearson correlation (x-axis) and Random Forest importance (%IncMSE, y-axis). Point size reflects evidence counts (number of contrasts in which the gene is differentially expressed), and color indicates the direction of correlation (positive or negative). Top-ranking genes are labeled. (**B**) Density distribution of adjusted correlation values stratified by evidence level, demonstrating that genes supported by multiple contrasts tend to exhibit stronger and more consistent associations with the emulsification phenotype. (**C**) Relationship between absolute correlation (|r|) and Random Forest importance (%IncMSE), illustrating concordance between linear association and non-linear predictive contribution. The dashed line represents a linear regression fit.

Integration of these three analytical layers yielded a consensus set of 14 prioritized candidate genes (Table S2), each supported by multiple statistical criteria. Feature selection was constrained by sample size (*n* = 18) to reduce overfitting. Because the transcriptomic dataset included 18 samples, the machine learning analysis should be interpreted as a hypothesis-generating prioritization approach rather than as a definitive predictive model. Correlation coefficients and Random Forest importance values indicate statistical association with the emulsification phenotype, but they do not demonstrate mechanistic involvement. Therefore, the prioritized genes should be considered candidates for future functional validation. The final Random Forest model, trained on residualized data, achieved an R2 of ~0.58–0.63. The model parameters were optimized by minimizing the out-of-bag (OOB) mean squared error, resulting in an mtry of 2–3, which aligns with standard heuristics for our constrained feature space (≤14 genes), and a ntree of 500. This performance reflects predictive capacity within the limits of the dataset and contrasts with the performance observed prior to adjustment (R^2^ ≈ 0.85), suggesting that the refined model captured phenotype-associated signals after reducing condition- and time-related confounding. The integrative relationships among correlation, predictive importance, and evidence support are summarized in Figure 7. The multi-dimensional analysis (Figure 7A) reveals concordance between correlation strength and Random Forest importance, indicating agreement between linear and non-linear modeling approaches. Highly ranked genes consistently displayed strong positive or negative correlations with E24, supporting their prioritization as candidates for future functional validation rather than confirming their mechanistic involvement. Notably, both positively and negatively associated genes were represented, suggesting that the emulsification phenotype may be linked to a broader transcriptional network involving both increased and decreased gene expression patterns. To further prioritize candidate genes, a composite ranking score integrating correlation magnitude and Random Forest importance was calculated (Figure 7B). This approach highlights a subset of top-ranked genes, including *ACR0PX_RS09150*, encoding a D-amino acid dehydrogenase, which exhibited a strong positive association with emulsification activity (r ≈ 0.81) and high predictive importance. This enzyme is annotated as involved in redox metabolism and amino acid catabolism, suggesting a possible association between cellular metabolic state, reducing power availability, and the emulsification phenotype. In contrast, genes such as *ACR0PX_RS16435* and *ACR0PX_RS15710*, which display strong negative correlations (r < −0.80) and high importance scores, may represent candidates associated with functions that vary inversely with emulsification activity. The distribution of correlation values across evidence levels (Figure 7C) further suggests that genes supported by multiple contrasts tended to exhibit stronger and more consistent associations with the phenotype, supporting the utility of the integrative framework. Additionally, the relationship between absolute correlation and RF importance shows a positive trend, suggesting that genes with stronger phenotypic associations also contribute more substantially to predictive performance.

Collectively, the integrative analysis highlighted a subset of genes statistically associated with the emulsification phenotype across multiple analytical approaches. The convergence of differential expression, correlation, and machine learning methods provided a useful framework for prioritizing candidate genes under inorganic carbon conditions.

## 3. Discussion

The present study demonstrates that inorganic carbon availability modulates emulsification activity and transcriptional responses in *Vreelandella zhaodongensis* BS253. The strain remained viable under oxygen-limited and inorganic carbon-enriched conditions and exhibited marked differences in emulsification activity depending on medium composition, CO_2_ concentration, and cultivation time. These results indicate that emulsification-associated responses in BS253 are condition-dependent and that distinct inorganic carbon regimes affect both the magnitude and temporal dynamics of the E24 phenotype [21].

The physiological behavior observed for BS253 is consistent with the metabolic plasticity expected from haloalkaliphilic bacteria inhabiting saline–alkaline environments, which are characterized by fluctuations in salinity, pH, oxygen availability, and dissolved inorganic carbon [22,23]. Such variability selects for microorganisms capable of adjusting membrane-associated processes and extracellular metabolite production [10,24,25]. In this context, the ability of BS253 to grow under oxygen limitation and under CO_2_- and bicarbonate-enriched conditions reinforces its ecological adaptability. Similar metabolic flexibility has been reported in carbon-responsive bacteria capable of utilizing inorganic carbon sources to sustain the extracellular emulsification or biosurfactant-associated phenotypes. For instance, *Bacillus* sp. ISTS2 was shown to produce biosurfactants under both CO_2_-enriched atmospheres and bicarbonate supplementation, although the highest yields were still observed in the presence of organic substrates such as n-hexadecane [26]. These findings suggest that inorganic carbon can support emulsification-associated phenotypes, even if it does not always maximize them, a pattern that aligns with the behavior observed in BS253.

A key observation in this study is that CO_2_ and NaHCO_3_ do not produce equivalent physiological responses. While NaHCO_3_ supplementation promoted higher biomass accumulation and sustained absolute emulsification activity, CO_2_ enrichment resulted in lower biomass but higher emulsification relative to cell density. The reduced activity observed under 20% CO_2_, followed by partial recovery after extended cultivation or increased inoculum density, suggests that elevated CO_2_ may initially impose physiological constraints on BS253. Although the mechanism was not directly tested, this response may be related to acid–base imbalance, CO_2_-associated stress, altered growth dynamics, or delayed acclimation. The partial recovery observed over time or at higher initial cell density may reflect physiological adaptation or improved buffering capacity of denser cultures. From a process-development perspective, these results suggest that NaHCO_3_ may be preferable when biomass accumulation is prioritized, whereas moderate CO_2_ enrichment may favor higher emulsification activity relative to biomass. CO_2_ concentration, inoculum density, and cultivation time should therefore be considered key variables in future BS253 optimization and scale-up studies. Together, these results support the view that inorganic carbon availability influences both growth and emulsification-associated responses, with outcomes depending on the chemical form and concentration of the carbon source [27,28,29,30].

Previous studies using *Bacillus* spp. have reported enhanced biosurfactant production under bicarbonate supplementation, particularly in systems with carbon concentration mechanisms, where bicarbonate serves as an accessible substrate for anaplerotic pathways. For example, in *Bacillus* sp. SS105, bicarbonate-driven cultivation resulted in substantial biosurfactant production, with yields strongly influenced by cultivation parameters such as bicarbonate concentration and incubation time. However, these studies largely focused on endpoint production metrics [31]. In contrast, the present work demonstrates that different inorganic carbon sources can differentially affect growth-associated versus specific production profiles, highlighting a more nuanced relationship between carbon availability and metabolic allocation. The contrasting results obtained in the original saline medium and in MSM further indicate that the emulsification phenotype is conditional rather than constitutive. In the original saline medium, 5% CO_2_ did not result in detectable E24, whereas MSM supported high activity under CO_2_ and NaHCO_3_ supplementation. This difference may reflect changes in nutrient availability, ionic composition, pH buffering capacity, or metabolic allocation. Therefore, CO_2_ should not be viewed as an isolated inducer of emulsification activity but as one component of a broader cultivation context that modulates extracellular responses in BS253.

These phenotypic differences were reflected at the transcriptional level. Inorganic carbon availability was associated with broad transcriptional changes, consistent with the central role of carbon metabolism in bacterial regulatory networks [32,33]. The transition from 12 h to 18 h likely represents a shift in cellular physiological state, as commonly observed during growth phase-dependent transcriptional responses [34]. The higher number of differentially expressed genes under NaHCO_3_ supplementation indicates a more extensive regulatory response compared to CO_2_ under the tested conditions. Rather than pointing to a single biosynthetic pathway, the data support a distributed response involving transport systems, stress adaptation, membrane-associated functions, and extracellular processes. This systems-level response contrasts with earlier studies that emphasized specific enzymatic pathways, such as carbonic anhydrase or PEP carboxylase activity, as indicators of inorganic carbon utilization, and instead highlights the association between the emulsification phenotype and broader cellular networks [34,35,36,37].

Functional enrichment analyses further support the involvement of transport and membrane-associated processes in the emulsification phenotype. The enrichment of ABC-type transporters and ATPase-coupled transmembrane systems suggests a role in the export, assembly, or regulation of extracellular compounds [38,39,40]. Such systems are known to participate in secretion and envelope remodeling, particularly under saline and alkaline stress conditions. This supports the hypothesis that the emulsification phenotype in BS253 is associated with a broader adaptive response involving membrane-associated functions, transport systems, and extracellular processes, rather than with a single confirmed biosynthetic module [41,42].

This broader interpretation is reinforced by the integrative analytical approach used in this study, combining differential expression, phenotypic correlation, and machine learning-based prioritization. In contrast to studies relying solely on differential expression, this approach identifies genes consistently associated with the phenotype across multiple conditions, which is particularly important in multifactorial systems [43,44]. Although functional validation will be required to determine the direct role of these candidate genes, the convergence among independent analytical methods strengthens their relevance and provides a more robust framework for future mechanistic studies.

The identification of genes associated with EPS precursor synthesis, glycosyltransferase activity, and polysaccharide export suggests a possible EPS-related basis for the emulsification phenotype. This interpretation is consistent with reports that microbial EPS can function as emulsifying agents [45,46], and the absence of substantial surface tension reduction in BS253 suggests that the emulsification-active material does not behave as a classical low-molecular-weight biosurfactant [8,47]. Preliminary qualitative biochemical screening of purified and lyophilized extracellular fractions further indicated carbohydrate-associated and amino-containing components, anionic functional groups, and limited neutral lipid-associated signals. Together, these observations are compatible with a complex extracellular polymeric fraction, but they do not establish EPS identity, chemical composition, or biosynthetic origin. Therefore, further purification, quantitative compositional analysis, and structural validation will be required to define the active material.

For the stability assays, the active preparation was solubilized and filtered through 0.22 µm membranes before E24 determination, reducing the potential contribution of intact cells and larger cell-associated particles. However, this procedure does not provide chemical purification or compositional identification. In addition, full characterization of the extracellular emulsification-active material is technically challenging because the active fraction was obtained from a complex extracellular preparation, which may interfere with purification and compositional analysis. Such characterization generally requires multiple sequential optimization steps, including extraction, fractionation, recovery of the active fraction, and validation of emulsification activity after each purification step. These procedures are time-consuming and often require iterative trial-and-error adjustments to preserve activity while removing interfering components. Therefore, although E24 is a useful initial screening parameter, future purification, EPS quantification, and biochemical and structural analyses will be required to determine the chemical composition, biosynthetic origin, and functional properties of the emulsification-active material.

The integration of machine learning approaches enabled the prioritization of a subset of genes statistically associated with the emulsification phenotype. Among these, *ACR0PX_RS09150*, encoding a D-amino acid dehydrogenase, emerged as a top-ranking candidate, showing a strong positive correlation with emulsification activity. This enzyme is annotated as involved in amino acid metabolism and redox balance, suggesting a possible association between cellular metabolic state and the emulsification phenotype. In other microbial models, *dadA*, which encodes a D-amino acid dehydrogenase, has been associated with biofilm matrix regulation. For example, in *Azospirillum brasilense* Sp7, the transcription of *dadA* is regulated by the transcriptional activator TyrR, which also participates in the regulation of biofilm formation and EPS production [48]. Furthermore, D-amino acid homeostasis is vital for cell stiffness and peptidoglycan alteration. In *Pseudomonas aeruginosa*, disruption of *dadA* has been associated with intracellular accumulation of D-alanine, altered peptidoglycan cross-linking, and impaired formation of robust biofilms containing EPS components such as alginate [49]. In *V. zhaodongensis* BS253, the strong positive association of this dehydrogenase with the emulsification phenotype (E24) suggests that *ACR0PX_RS09150* may represent a relevant candidate for future studies addressing whether D-amino acid metabolism, redox balance, or envelope-associated processes contribute to extracellular emulsification-associated responses under inorganic carbon enrichment. These findings provide a hypothesis-generating framework in which core metabolism may be linked to extracellular emulsification-associated responses. However, direct functional involvement of *ACR0PX_RS09150* or other prioritized genes will require genetic, enzymatic, or targeted metabolite validation.

An important strength of this study is the demonstration that the extracellular emulsification-active material obtained from BS253 remains active under a wide range of physicochemical conditions, including high salinity, temperature, pH, and pressure. While previous studies on CO_2_-responsive biosurfactant or emulsification-associated phenotypes have focused primarily on yield optimization and enzymatic characterization, the present work highlights the robustness of the extracellular material itself [50,51]. This feature is particularly relevant for applications in saline–alkaline environments, where process conditions are often harsh and may compromise the performance of conventional surface-active compounds [23,27,52]. Given the haloalkaliphilic nature of BS253, future studies should evaluate pH values above 10 to define the upper alkaline stability range of the extracellular emulsification-active material.

Overall, the results support a model in which inorganic carbon availability reshapes the physiology of BS253 and influences emulsification-associated responses through coordinated changes in growth behavior, membrane-associated functions, transport systems, and EPS-related pathways. The distinct responses observed under CO_2_ and bicarbonate conditions provide a framework for understanding how different inorganic carbon sources can be used to optimize emulsification-associated responses. Future studies should focus on the functional validation of the prioritized candidate genes, structural characterization of the extracellular emulsification-active material, and evaluation of low-cost CO_2_-rich industrial gas streams to further support the development of BS253-based bioprocesses.

## 4. Materials and Methods

### 4.1. Microorganism and Culture Conditions

The BS253 strain of *Vreelandella zhaodongensis* was previously isolated and routinely maintained in a saline medium [53,54]. To assess growth under anaerobic conditions, selected colonies were transferred to fresh saline agar plates and incubated in an anaerobic jar (Permution^®^ (Curitiba, Brazil), model JA0403) using a gas-generating system (GENbox Anaer, bioMérieux^®^, Marcy-l’Étoile, France). To evaluate the effect of inorganic carbon supplementation on growth, strain BS253 was cultivated on a complex mineral salt medium (MSM) that provides organic carbon, consisting of (per liter) 5 g yeast extract, 5 g NaCl, 5.7 g KH_2_PO_4_, 5.25 g K_2_HPO_4_, 3 g NaNO_3_, 1 g KCl, 0.51 g MgSO_4_·7H_2_O, 0.21 g CaCl_2_·2H_2_O, and 0.20 g Na_2_S_2_O_3_ [55,56]. Plates were incubated at 28 °C in a CO_2_-controlled incubator (New Brunswick™, Somerset, NJ, USA) under a 5% CO_2_ atmosphere, either with or without supplementation of 2% NaHCO_3_. Growth was monitored for up to 21 days.

### 4.2. Screening for Emulsification Activity

To evaluate culture conditions associated with emulsification activity, strain BS253 was initially cultivated in saline broth for 24 h. Cells were harvested by centrifugation (10,000 rpm, 10 min) (ROTINA 380 R, Hettich Zentrifugen, Tuttlingen, Germany), washed twice with sterile 0.9% NaCl solution, and resuspended in MSM. The inoculum was standardized to an optical density at 600 nm (OD_600_) of 1 or 5. Cultures were incubated at 28 °C for 72 h under agitation (180 rpm). An initial screening of emulsification activity was performed in the original saline medium under both aerobic conditions and a 5% CO_2_-enriched atmosphere. Subsequently, cultures were tested in MSM supplemented with 2% NaHCO_3_ under aerobic conditions or incubated under CO_2_-enriched atmospheres containing 5%, 10%, or 20% CO_2_. Emulsification activity was assessed using the E24 assay [57]. Briefly, 2 mL of kerosene (Química Borel Ltda., Porto Alegre, Brazil) was thoroughly mixed with an equal volume of culture for 2 min. After 24 h, the emulsion formed was calculated as the ratio of the emulsion layer height to the total liquid height. Surface tension was measured using the ring method [58]. Sodium dodecyl sulfate (SDS, 0.5%) was used as a positive control. All experiments were performed in biological triplicate. Statistical analyses were performed using GraphPad Prism (version 10.4.2).

### 4.3. Physicochemical Stability Assays

The stability of the emulsification activity was assessed by exposing the cell-free supernatant to different salinity, temperature, pH, and pressure conditions. Strain BS253 was cultivated in MSM broth under 5% CO_2_ at 28 °C with agitation for 72 h. Cells were harvested by centrifugation, washed twice with alkaline buffer (pH 9), and the putative extracellular material was solubilized under agitation and heating. The resulting cell-free preparation was then filtered through 0.22 µm membranes before exposure to the different physicochemical treatments and subsequent E24 determination. Thermal stability was evaluated by incubating the filtered samples at 4, 25, 37, 50, 75, and 100 °C. Salinity tolerance was tested at NaCl concentrations of 2, 5, 10, 20, and 30%. The effect of pH was evaluated at pH 2, 7, and 10 using sterile 1 M HCl or 1 M NaOH [59,60]. Resistance to severe processing conditions was evaluated by exposing the samples to combined high temperature and pressure (121 °C, 2 atm, 15 min). E24 was subsequently measured to determine compound stability after each treatment.

### 4.4. Preliminary Qualitative Biochemical Screening of Extracellular Fractions

Preliminary qualitative biochemical screening of the extracellular fraction was performed to assess the presence of major biochemical classes, including carbohydrate-associated components, amino-containing compounds, anionic functional groups, and neutral lipid-associated moieties. Extracellular material was recovered after alkaline extraction and filtration through 0.22 µm membranes. The filtered material was then processed by ethanol precipitation, dialysis using a 3 kDa molecular weight cutoff membrane (Sigma-Aldrich, St. Louis, MO, USA), and lyophilization. In parallel, aliquots were concentrated using Amicon (Miami, FL, USA) ultrafiltration units with a 3.7 kDa cutoff and further fractionated by size-exclusion chromatography on a Sephadex LH-20 column (Cytiva, Marlborough, MA, USA). The assays were based on color development and/or precipitate formation using appropriate positive and negative controls. Carbohydrate-associated components were evaluated by the orcinol reaction, amino-containing compounds by the ninhydrin assay, anionic functional groups by CTAB/methylene blue complexation, and neutral lipid-associated components by iodine/potassium iodide staining.

### 4.5. Time-Course Analysis of Emulsification Activity

To investigate the temporal dynamics of emulsification activity, strain BS253 was cultivated in MSM and sampled at different time points (2, 6, 12, 15, 18, 24, and 48 h) to monitor both cell growth and emulsification activity. Microbial growth was assessed by measuring OD_600_, whereas emulsification activity was evaluated using the E24. These time-course data were used to identify physiologically informative stages for transcriptomic analysis.

### 4.6. Transcriptomic Analysis

To investigate transcriptional responses associated with inorganic carbon availability and emulsification activity, strain BS253 was cultivated under three experimental conditions: (i) basal MSM (which provides organic carbon via yeast extract) without additional inorganic carbon supplementation (control), (ii) MSM under 10% CO_2_-enriched atmosphere (CO_2_), and (iii) MSM supplemented with 2% NaHCO_3_ (NaHCO_3_). The cultures were incubated at 28 °C under 180 rpm. Samples were collected at two cultivation time points (12 h and 18 h), with three biological replicates per condition. The time points were selected based on growth dynamics and emulsification profiles. Cells were suspended in RNAlater and sent for RNA isolation, and library preparation and sequencing were performed at the LaCTAD facility (www.lactad.unicamp.br, accessed on 3 June 2026) using an Illumina (San Diego, CA, USA) NextSeq 2000 platform. Raw reads were quality-checked using FastQC (v 0.12.1) and processed with fastp (v 1.3.3) for adapter trimming and filtering [61]. High-quality reads were aligned to the *V. zhaodongensis* BS253 reference genome (NCBI accession GCF_051175405.1) using Bowtie2 (v2.5.5) [62]. Alignment files were converted to BAM format and sorted using samtools (v 1.23.1) [63]. Gene counts were generated using featureCounts of the Subread package (v2.1.1), and differential gene expression analysis was performed using the DESeq2 package (v 1.52.0) [64,65]. Genes were considered differentially expressed when the FDR-adjusted *p*-value was <0.05, and the absolute log_2_ fold change was greater than 1. To identify biological processes associated with transcriptional changes, Gene Ontology (GO) enrichment analysis was conducted using EnrichR (v 3.4) [66]. The predicted proteome of strain BS253 was retrieved from the NCBI database using the Datasets tool (v 18.11.0) [67] and functionally annotated against the KEGG database [68], as well as the eggNOG Database using eggnog-mapper (v 2.1.13). Custom bash and R scripts are available in Supplementary Files S1 and S2, respectively.

### 4.7. Integrative Identification of Candidate Genes Associated with Emulsification

To identify genes associated with the emulsification phenotype, an integrative framework was applied. It combined differential expression evidence, phenotype association, and machine learning-based modeling. Differentially expressed genes (DEGs) identified across all experimental contrasts (FDR < 0.05; |log_2_FC| > 1) were used as input for integrative analysis. For each gene, an evidence score was calculated as the number of contrasts in which it was significantly differentially expressed. Gene expression values were obtained from variance-stabilized counts (DESeq2). To control for experimental effects, both expression data and emulsification values were adjusted using linear models accounting for condition and time. Residuals from these models (expression~condition + time; E24~condition + time) were used for downstream analyses. Pearson correlation coefficients were calculated between residualized gene expression profiles and residualized E24 values using complete observations. Genes with evidence ≥2 were retained and ranked based on the absolute value of the adjusted correlation coefficients. To avoid overfitting, the number of predictors included in the model was limited according to sample size (*n* = 18), with a maximum of 80% of samples (≤14 genes). The top-ranked genes were selected for modeling. Random Forest regression was performed using the package in R. Models were trained using residualized gene expression values as predictors and residualized E_24_ as the response variable. The mtry parameter was optimized by minimizing out-of-bag (OOB) mean squared error across a range of tested values. The final model was trained with 500 trees (*ntree* = 500). Variable importance was assessed using the percentage increase in mean squared error (%IncMSE). Final candidate genes were defined based on combined support from (i) evidence score, (ii) adjusted correlation with E24, and (iii) Random Forest importance. All analyses were performed in R (version 4.5.1) using custom scripts, available as Supplementary File S2.

## 5. Conclusions

Inorganic carbon availability influenced emulsification activity and transcriptional responses in *Vreelandella zhaodongensis* BS253, resulting in distinct physiological profiles according to the carbon source. The integration of E24 measurements and RNA-seq data indicates that the emulsification phenotype is associated with broader changes in membrane-associated functions, transport systems, and genes annotated as related to extracellular polymer biosynthesis. However, the chemical identity of the emulsification-active material and the functional roles of the prioritized genes remain to be experimentally validated. The stability of the extracellular activity across a wide range of physicochemical conditions highlights the potential relevance of BS253 for future studies on robust extracellular emulsification-active materials in saline–alkaline bioprocesses. Overall, this work provides a physiological and transcriptomic framework for future biochemical, genetic, and structural characterization of inorganic carbon-associated emulsification responses in haloalkaliphilic bacteria.

## Data Availability

The data supporting the findings of this study are available within the article and its Supplementary Materials. The minimal dataset has been provided as Supplementary Materials. Additional data are publicly available in the NCBI BioProject under accession number PRJNA1275882 (https://www.ncbi.nlm.nih.gov/bioproject/PRJNA1275882, accessed on 3 June 2026).

## Supplementary Materials

The following supporting information can be downloaded at: https://www.mdpi.com/article/10.3390/molecules31122182/s1, Figure S1: Growth of strain BS253 under anaerobiosis and 5% CO_2_-enriched conditions. Figure S2: Heatmap and hierarchical clustering of the top 100 most variable genes across experimental conditions and time points. Figure S3: Expression of key carbon metabolism genes. Figure S4. Transcriptional profiling of potential extracellular polysaccharide (EPS) biosynthetic genes in *V. zhaodongensis* BS253. Figure S5. Preliminary qualitative biochemical screening of the extracellular fraction. Table S1: Functional annotation, differential expression status, and emulsification correlation of exopolysaccharide (EPS)-related genes. Table S2: Prioritization of candidate genes associated with emulsification based on differential expression evidence, adjusted correlation, and Random Forest feature importance. File S1: Full bash script for file processing. File S2. Full R script for data processing and figure generation.

## References

[1] Gallo G., Aulitto M. (2024). Advances in Extremophile Research: Biotechnological Applications through Isolation and Identification Techniques. Life.

[2] De la Haba R.R., Arahal D.R., Sánchez-Porro C., Chuvochina M., Wittouck S., Hugenholtz P., Ventosa A. (2023). A long-awaited taxogenomic investigation of the family Halomonadaceae. Front. Microbiol..

[3] Margesin R., Schinner F. (2001). Potential of halotolerant and halophilic microorganisms for biotechnology. Extremophiles.

[4] Hobmeier K., Cantone M., Nguyen Q.A., Pflüger-Grau K., Kremling A., Kunte H.J., Pfeiffer F., Marin-Sanguino A. (2022). Adaptation to varying salinity in *Halomonas elongata*: Much more than ectoine accumulation. Front. Microbiol..

[5] Ma H., Zhao Y., Huang W., Zhang L., Wu F., Ye J., Chen G.-Q. (2020). Rational flux-tuning of *Halomonas bluephagenesis* for co-production of bioplastic PHB and ectoine. Nat. Commun..

[6] Kubicki S., Bollinger A., Katzke N., Jaeger K.E., Loeschcke A., Thies S. (2019). Marine biosurfactants: Biosynthesis, structural diversity and biotechnological applications. Mar. Drugs.

[7] Zhao F., Wu Y., Liu L. (2025). Enhanced anaerobic synthesis of rhamnolipids and emulsification of crude oil by genetically engineered *Pseudomonas aeruginosa* strains. Microb. Cell Fact..

[8] Sałek K., Euston S.R. (2019). Sustainable microbial biosurfactants and bioemulsifiers for commercial exploitation. Process Biochem..

[9] Ferreira I.N.S., Rodríguez D.M., Campos-Takaki G.M., Andrade R.F.d.S. (2020). Biosurfactant and bioemulsifier as promising molecules produced by *Mucor hiemalis* isolated from Caatinga soil. Electron. J. Biotechnol..

[10] Pardhi D.S., Panchal R.R., Raval V.H., Joshi R.G., Poczai P., Almalki W.H., Rajput K.N. (2022). Microbial surfactants: A journey from fundamentals to recent advances. Front. Microbiol..

[11] Eras-Muñoz E., Farré A., Sánchez A., Font X., Gea T. (2022). Microbial biosurfactants: A review of recent environmental applications. Bioengineered.

[12] Napp A.P., Dutra W.L., Duarte L.S., Abati E.V., dos Santos F.M., Melo C.L. (2026). CO_2_-driven biosurfactant synthesis by bacteria within CCUS. Appl. Microbiol. Biotechnol..

[13] Liu X., Li L., Zhao G., Xiong P. (2024). Optimization strategies for CO_2_ biological fixation. Biotechnol. Adv..

[14] Hu G., Li Y., Ye C., Liu L., Chen X. (2019). Engineering Microorganisms for Enhanced CO_2_ Sequestration. Trends Biotechnol..

[15] Laroche C. (2022). Exopolysaccharides from Microalgae and Cyanobacteria: Diversity of Strains, Production Strategies, and Applications. Mar. Drugs.

[16] Assine M.L., Vieira B.C., Salgado A.A.R., Santos L.J.C. (2015). Brazilian Pantanal: A Large Pristine Tropical Wetland. Landscapes and Landforms of Brazil.

[17] Louzada R.O., Bergier I., Roque F.O., McGlue M.M., Silva A., Assine M.L. (2021). Avulsions drive ecosystem services and economic changes in the Brazilian Pantanal wetlands. Curr. Res. Environ. Sustain..

[18] Hanifa M., Agarwal R., Sharma U., Thapliyal P.C., Singh L.P. (2023). A review on CO_2_ capture and sequestration in the construction industry: Emerging approaches and commercialised technologies. J. CO_2_ Util..

[19] Shaw R., Halder U., Mazumder K., Chaudhuri P., Bandopadhyay R. (2026). Characterisation and bioactivity of sulphated exopolysaccharide produce by *Halomonas titanicae* PCRB123b from Antarctic Ocean. Nat. Prod. Res..

[20] Kim B., Yang A.-I., Joe H.-I., Kim K.H., Choe H., Joe S.-H., Jun M.O., Shin N.-R. (2023). Genomic attributes and characterization of novel exopolysaccharide-producing bacterium *Halomonas piscis* sp. nov. isolated from jeotgal. Front. Microbiol..

[21] Choi K.R., Ahn Y.-J., Lee S.Y. (2022). Bacterial conversion of CO_2_ to organic compounds. J. CO_2_ Util..

[22] Mangoma N., Zhou N., Ncube T. (2025). Exploring the diversity and phenotypic properties of culturable haloalkaliphilic bacteria from soda pans in Buhera, Zimbabwe. Acad. Biol..

[23] Kanekar P.P., Kanekar S.P., Kanekar P.P., Kanekar S.P. (2022). Alkaliphilic, Alkalitolerant Microorganisms. Diversity and Biotechnology of Extremophilic Microorganisms from India.

[24] Narumi I., Ito M., Narumi I., Doukyu N. (2026). Diversity and Distribution of Extremophiles. Extremophiles for a Sustainable Future.

[25] Sigida E.N., Kuzina M.S., Kokoulin M.S., Ibrahim I.M., Grinev V.S., Konnova S.A., Fedonenko Y.P. (2024). Structure of the O-polysaccharide from the moderately halophilic bacterium *Halomonas fontilapidosi* KR26. Carbohydr. Res..

[26] Sundaram S., Thakur I.S. (2015). Biosurfactant production by a CO_2_ sequestering *Bacillus* sp. strain ISTS2. Bioresour. Technol..

[27] Kajla S., Kumari R., Nagi G.K. (2022). Microbial CO_2_ fixation and biotechnology in reducing industrial CO_2_ emissions. Arch. Microbiol..

[28] Kumar M., Sundaram S., Gnansounou E., Larroche C., Thakur I.S. (2018). Carbon dioxide capture, storage and production of biofuel and biomaterials by bacteria: A review. Bioresour. Technol..

[29] Hicks N., Vik U., Taylor P., Ladoukakis E., Park J., Kolisis F., Jakobsen K.S. (2017). Using Prokaryotes for Carbon Capture Storage. Trends Biotechnol..

[30] Onyeaka H., Ekwebelem O.C. (2023). A review of recent advances in engineering bacteria for enhanced CO_2_ capture and utilization. Int. J. Environ. Sci. Technol..

[31] Maheshwari N., Kumar M., Thakur I.S., Srivastava S. (2017). Recycling of carbon dioxide by free air CO_2_ enriched (FACE) *Bacillus* sp. SS105 for enhanced production and optimization of biosurfactant. Bioresour. Technol..

[32] Spona-Friedl M., Braun A., Huber C., Eisenreich W., Griebler C., Kappler A., Elsner M. (2020). Substrate-dependent CO_2_ fixation in heterotrophic bacteria revealed by stable isotope labelling. FEMS Microbiol. Ecol..

[33] Erb T. (2011). Carboxylases in Natural and Synthetic Microbial Pathways. Appl. Environ. Microbiol..

[34] López-Maury L., Marguerat S., Bähler J. (2008). Tuning gene expression to changing environments: From rapid responses to evolutionary adaptation. Nat. Rev. Genet..

[35] Pinu F.R., Villas-Boas S.G. (2017). Extracellular microbial metabolomics: The state of the art. Metabolites.

[36] Kochanowski K., Okano H., Patsalo V., Williamson J., Sauer U., Hwa T. (2021). Global coordination of metabolic pathways in *Escherichia coli* by active and passive regulation. Mol. Syst. Biol..

[37] Scott M., Gunderson C., Mateescu E., Zhang Z., Hwa T. (2010). Interdependence of Cell Growth and Gene Expression: Origins and Consequences. Science.

[38] Zhou Z., White K.A., Polissi A., Georgopoulos C., Raetz C.R.H. (1998). Function of *Escherichia coli* MsbA, an Essential ABC Family Transporter, in Lipid A and Phospholipid Biosynthesis. J. Biol. Chem..

[39] Locher K.P. (2004). Structure and mechanism of ABC transporters. Curr. Opin. Struct. Biol..

[40] Higgins C.F. (2001). ABC transporters: Physiology, structure and mechanism—An overview. Res. Microbiol..

[41] Sousa J.A.B., Sorokin D.Y., Bijmans M.F.M., Plugge C.M., Stams A.J.M. (2015). Ecology and application of haloalkaliphilic anaerobic microbial communities. Appl. Microbiol. Biotechnol..

[42] Dubey S., Shah F., Mishra S. (2026). Adaptation strategies in halophilic bacteria and its importance. Microbial Stress Survival.

[43] Tarca A.L., Bhatti G., Romero R. (2013). A Comparison of Gene Set Analysis Methods in Terms of Sensitivity, Prioritization and Specificity. PLoS ONE.

[44] Libbrecht M.W., Noble W.S. (2015). Machine learning applications in genetics and genomics. Nat. Rev. Genet..

[45] Nichols C.A.M., Guezennec J., Bowman J.P. (2005). Bacterial Exopolysaccharides from Extreme Marine Environments with Special Consideration of the Southern Ocean, Sea Ice, and Deep-Sea Hydrothermal Vents: A Review. Mar. Biotechnol..

[46] Paniagua-Michel J.d.J., Olmos-Soto J., Morales-Guerrero E.R., Kim S.-K. (2014). Chapter Eleven—Algal and Microbial Exopolysaccharides: New Insights as Biosurfactants and Bioemulsifiers. Advances in Food and Nutrition Research.

[47] Gutnick D., Shabtai Y. (2017). Exopolysaccharide Bioemulsifiers. Biosurfactants and Biotechnology.

[48] Jijón-Moreno S., Baca B.E., Castro-Fernández D.C., Ramírez-Mata A. (2019). TyrR is involved in the transcriptional regulation of biofilm formation and D-alanine catabolism in *Azospirillum brasilense* Sp7. PLoS ONE.

[49] Trivedi R.R., Crooks J.A., Auer G.K., Pendry J., Foik I.P., Siryaporn A., Abbott N.L., Gitai Z., Weibel D.B. (2018). Mechanical Genomic Studies Reveal the Role of D-Alanine Metabolism in *Pseudomonas aeruginosa* Cell Stiffness. mBio.

[50] Ghojavand H., Vahabzadeh F., Azizmohseni F. (2011). A halotolerant, thermotolerant, and facultative biosurfactant producer: Identification and molecular characterization of a bacterium and evolution of emulsifier stability of a lipopeptide biosurfactant. Biotechnol. Bioprocess Eng..

[51] Marchant R., Banat I. (2012). Biosurfactants: A sustainable replacement for chemical surfactants?. Biotechnol. Lett..

[52] Sarubbo L.A., Silva M.d.G.C., Durval I.J.B., Bezerra K.G.O., Ribeiro B.G., Silva I.A., Twigg M.S., Banat I.M. (2022). Biosurfactants: Production, properties, applications, trends, and general perspectives. Biochem. Eng. J..

[53] Lautert-Dutra W., dos Santos F.M., Pasinato Napp A., Lovato Melo C. (2025). Draft genome sequence of *Vreelandella stevensii* strain BS235 isolated from hypersaline lakes from Brazilian pantanal. Microbiol. Resour. Announc..

[54] Poli A., Esposito E., Orlando P., Lama L., Giordano A., de Appolonia F., Nicolaus B., Gambacorta A. (2007). *Halomonas alkaliantarctica* sp. nov.; isolated from saline lake Cape Russell in Antarctica, an alkalophilic moderately halophilic, exopolysaccharide-producing bacterium. Syst. Appl. Microbiol..

[55] Liang X., Shi R., Radosevich M., Zhao F., Zhang Y., Han S., Zhang Y. (2017). Anaerobic lipopeptide biosurfactant production by an engineered bacterial strain for in situ microbial enhanced oil recovery. RSC Adv..

[56] Mishra S., Gupta S., Raghuvanshi S., Pal P. (2016). Energetic assessment of fixation of CO_2_ and subsequent biofuel production using *B. cereus* SM1 isolated from sewage treatment plant. Bioprocess Biosyst. Eng..

[57] Cooper D.G., Goldenberg B.G. (1987). Surface-Active Agents from Two *Bacillus* Species. Appl. Environ. Microbiol..

[58] Brito H.A., Napp A.P., Pereira E., Bach E., Borowski J.V.B., Passaglia L.M.P., Melo V.M.M., Moreira R., Foster E.J., Lopes F.C. (2024). Enhanced low-cost lipopeptide biosurfactant production by *Bacillus velezensis* from residual glycerin. Bioprocess Biosyst. Eng..

[59] Moro G.V., Almeida R.T., Napp A.P., Porto C., Pilau E.J., Lüdtke D.S., Moro A.V., Vainstein M.H. (2018). Identification and ultra-high-performance liquid chromatography coupled with high-resolution mass spectrometry characterization of biosurfactants, including a new surfactin, isolated from oil-contaminated environments. Microb. Biotechnol..

[60] Abdel-Mawgoud A.M., Aboulwafa M.M., Hassouna N.A.-H. (2008). Characterization of Surfactin Produced by *Bacillus subtilis* Isolate BS5. Appl. Biochem. Biotechnol..

[61] Chen S., Zhou Y., Chen Y., Gu J. (2018). fastp: An ultra-fast all-in-one FASTQ preprocessor. Bioinformatics.

[62] Langfelder P., Horvath S. (2008). WGCNA: An R package for weighted correlation network analysis. BMC Bioinform..

[63] Danecek P., Bonfield J.K., Liddle J., Marshall J., Ohan V., Pollard M.O., Whitwham A., Keane T., McCarthy S.A., Davies R.M. (2021). Twelve years of SAMtools and BCFtools. Gigascience.

[64] Liao Y., Smyth G.K., Shi W. (2014). featureCounts: An efficient general purpose program for assigning sequence reads to genomic features. Bioinformatics.

[65] Love M.I., Huber W., Anders S. (2014). Moderated estimation of fold change and dispersion for RNA-seq data with DESeq2. Genome Biol..

[66] Kuleshov M.V., Jones M.R., Rouillard A.D., Fernandez N.F., Duan Q., Wang Z., Koplev S., Jenkins S.L., Jagodnik K.M., Lachmann A. (2016). Enrichr: A comprehensive gene set enrichment analysis web server 2016 update. Nucleic Acids Res..

[67] O’lEary N.A., Cox E., Holmes J.B., Anderson W.R., Falk R., Hem V., Tsuchiya M.T.N., Schuler G.D., Zhang X., Torcivia J. (2024). Exploring and retrieving sequence and metadata for species across the tree of life with NCBI Datasets. Sci. Data.

[68] Kanehisa M., Furumichi M., Tanabe M., Sato Y., Morishima K. (2016). KEGG: New perspectives on genomes, pathways, diseases and drugs. Nucleic Acids Res..

