# Supplementary material for: Inorganic Carbon Modulates Emulsification Activity and Transcriptional Responses in Vreelandella zhaodongensis BS253
Source: Molecules. 2026 Jun 22;31(12):2182. doi: 10.3390/molecules31122182 (PMC13305022; doi:10.3390/molecules31122182)
Supplement: Supplementary file 1 [file molecules-31-02182-s001.zip › File_S1.sh.pdf]

```
#!/bin/bash

#
=========================================================================
====
# COMPLETE RNA-SEQ ANALYSIS PIPELINE
#
=========================================================================
====
# This script performs:
#   1. Download reference genome, GFF, and proteome from NCBI
#   2. Quality control and trimming with fastp
#   3. Read mapping with Bowtie2
#   4. Gene counting with featureCounts
#   5. Functional annotation with EggNOG-mapper
#
=========================================================================
====

set -e  # Stop script on error
set -u  # Stop on undefined variables

#
=========================================================================
====
# CONFIGURATION
#
=========================================================================
====

# Number of CPU threads to use
THREADS=8

# NCBI accession for reference data
NCBI_ACCESSION="GCF_051175405.1"

# Directory structure
RAW_DATA_DIR="."
QC_REPORTS_DIR="qc_reports"
TRIMMED_DIR="trimmed_data"
GENOME_DIR="genome"
MAPPING_DIR="mapping"
COUNTS_DIR="counts"
EGGNOG_DIR="eggnog_annotation"

# Reference files
GENOME_FASTA="${GENOME_DIR}/genomic.fna"
GENOME_GFF="${GENOME_DIR}/genomic.gff"
PROTEOME_FASTA="${GENOME_DIR}/protein.faa"
GENOME_IDX="${GENOME_DIR}/bowtie2_index"

# Create output directories
mkdir -p $QC_REPORTS_DIR $TRIMMED_DIR $MAPPING_DIR $COUNTS_DIR
$EGGNOG_DIR

#
=========================================================================
====
```

```
# STEP 0: SETUP CONDA ENVIRONMENTS
#
=========================================================================
====

echo ">> Creating conda environment for FastQC..."
conda create -n fastqc_env -c bioconda fastqc -y

echo ">> Creating conda environment for fastp..."
conda create -n fastp_env -c bioconda fastp -y

echo ">> Creating conda environment for Bowtie2..."
conda create -n bowtie2_env -c bioconda bowtie2 -y

echo ">> Creating conda environment for Samtools..."
conda create -n samtools_env -c bioconda samtools -y

echo ">> Creating conda environment for featureCounts (subread
v2.1.1)..."
conda create -n subread_env -c bioconda subread=2.1.1 -y

echo ">> Creating conda environment for EggNOG-mapper v2.1.12..."
conda create -n eggnog_env -c bioconda eggnog-mapper=2.1.12 -y

echo ">> Creating conda environment for NCBI datasets..."
conda create -n ncbi_env -c conda-forge ncbi-datasets-cli -y


#
=========================================================================
====
# STEP 1: DOWNLOAD REFERENCE DATA FROM NCBI
#
=========================================================================
====

# Download genome, GFF, and proteome using NCBI datasets

conda run -n ncbi_env datasets download genome accession
${NCBI_ACCESSION} \
    --include genome,gff,protein \
    --filename ${GENOME_DIR}/reference_data.zip

# Extract downloaded files
if [ -f "${GENOME_DIR}/reference_data.zip" ]; then
    echo ">> Extracting reference data..."
    unzip -o ${GENOME_DIR}/reference_data.zip -d ${GENOME_DIR}/

    # Find and rename the extracted files
    find ${GENOME_DIR}/ncbi_dataset/data/${NCBI_ACCESSION}/ -name "*.fna"
-exec cp {} ${GENOME_FASTA} \;
    find ${GENOME_DIR}/ncbi_dataset/data/${NCBI_ACCESSION}/ -name "*.gff"
-exec cp {} ${GENOME_GFF} \;
    find ${GENOME_DIR}/ncbi_dataset/data/${NCBI_ACCESSION}/ -name "*.faa"
-exec cp {} ${PROTEOME_FASTA} \;

    # Clean up
    rm ${GENOME_DIR}/reference_data.zip
```

```
    echo "âœ" Reference files downloaded and extracted"
else
    echo "ERROR: Failed to download reference data"
    exit 1
fi

# Verify files exist
if [ ! -f "${GENOME_FASTA}" ] || [ ! -f "${GENOME_GFF}" ] || [ ! -f
"${PROTEOME_FASTA}" ]; then
    echo "ERROR: Reference files not found after extraction"
    echo "Please check the NCBI dataset structure and adjust paths"
    exit 1
fi

echo "âœ" Genome: ${GENOME_FASTA}"
echo "âœ" GFF annotation: ${GENOME_GFF}"
echo "âœ" Proteome: ${PROTEOME_FASTA}"
echo ""

#
========================================================================
====
# STEP 2: INITIAL QUALITY CONTROL (FastQC)
#
========================================================================
====

echo ">> Running FastQC on raw FASTQ files..."
conda run -n fastqc_env fastqc ${RAW_DATA_DIR}/*.fastq.gz -o
$QC_REPORTS_DIR -t $THREADS

echo "âœ" FastQC analysis complete. Reports in: ${QC_REPORTS_DIR}"
echo ""

#
========================================================================
====
# STEP 3: READ TRIMMING AND FILTERING WITH fastp
#
========================================================================
====
echo
"=======================================================================
========"
echo "STEP 3: READ TRIMMING AND QUALITY FILTERING WITH FASTP"
echo
"=======================================================================
========"

# Process each pair of FASTQ files
for R1 in ${RAW_DATA_DIR}/*_R1_001.fastq.gz; do
    # Extract base sample name
    SAMPLE_NAME=$(basename $R1 _R1_001.fastq.gz)
    R2=${RAW_DATA_DIR}/${SAMPLE_NAME}_R2_001.fastq.gz

    echo ">> Processing sample: ${SAMPLE_NAME}"

    conda run -n fastp_env fastp \
```

```
        -i $R1 \
        -I $R2 \
        -o ${TRIMMED_DIR}/${SAMPLE_NAME}.R1.trimmed.fastq.gz \
        -O ${TRIMMED_DIR}/${SAMPLE_NAME}.R2.trimmed.fastq.gz \
        -w $THREADS \


    echo "    âœ" Trimmed reads saved to:
${TRIMMED_DIR}/${SAMPLE_NAME}.*.trimmed.fastq.gz"
done

echo "âœ" Trimming complete"
echo ""

#
=========================================================================
====
# STEP 4: BUILD BOWTIE2 INDEX
#
=========================================================================
====
echo
"========================================================================
========"
echo "STEP 4: BUILDING BOWTIE2 INDEX FOR REFERENCE GENOME"
echo
"========================================================================
========"

if [ ! -f "${GENOME_IDX}.1.bt2" ]; then
    echo ">> Building Bowtie2 index..."
    conda run -n bowtie2_env bowtie2-build --threads $THREADS
$GENOME_FASTA $GENOME_IDX
    echo "âœ" Bowtie2 index built"
else
    echo "âœ" Bowtie2 index already exists, skipping..."
fi
echo ""

#
=========================================================================
====
# STEP 5: READ MAPPING, CONVERSION, AND COUNTING
#
=========================================================================
====
echo
"========================================================================
========"
echo "STEP 5: READ MAPPING, BAM CONVERSION, AND FEATURE COUNTING"
echo
"========================================================================
========"

for R1_TRIMMED in ${TRIMMED_DIR}/*.R1.trimmed.fastq.gz; do
    # Extract base sample name
    SAMPLE_NAME=$(basename $R1_TRIMMED .R1.trimmed.fastq.gz)
    R2_TRIMMED=${TRIMMED_DIR}/${SAMPLE_NAME}.R2.trimmed.fastq.gz
```

```
    echo "------------------------------------------------------"
    echo "Processing sample: ${SAMPLE_NAME}"
    echo "------------------------------------------------------"

    # Define intermediate file names
    SAM_FILE="${MAPPING_DIR}/${SAMPLE_NAME}.sam"
    BAM_FILE="${MAPPING_DIR}/${SAMPLE_NAME}.sorted.bam"
    COUNT_FILE="${COUNTS_DIR}/${SAMPLE_NAME}.txt"

    # Step 5a: Alignment with Bowtie2
    echo "--> Step 5a/4: Aligning with Bowtie2..."
    conda run -n bowtie2_env bowtie2 -x $GENOME_IDX \
        -1 $R1_TRIMMED \
        -2 $R2_TRIMMED \
        -S $SAM_FILE \
        -p $THREADS \
        --very-sensitive \
        --no-mixed \
        --no-discordant

    # Check if alignment was successful
    if [ $? -eq 0 ]; then
        # Step 5b: Convert SAM to sorted BAM
        echo "--> Step 5b/4: Converting and sorting with Samtools..."
        conda run -n samtools_env samtools view -bS $SAM_FILE | conda run
-n samtools_env samtools sort -o $BAM_FILE -@ $THREADS

        # Index the BAM file
        conda run -n samtools_env samtools index $BAM_FILE

        # Step 5c: Count reads with featureCounts v2.1.1
        echo "--> Step 5c/4: Counting reads with featureCounts v2.1.1..."
        conda run -n subread_env featureCounts \
            -T $THREADS \
            -p \
            -t CDS \
            -g locus_tag \
            -a ${GENOME_GFF} \
            -o ${COUNT_FILE} \
            ${BAM_FILE}

        # Step 5d: Cleanup intermediate files
        echo "--> Step 5d/4: Cleaning up intermediate files..."
        rm $SAM_FILE

        echo "âœ" Sample ${SAMPLE_NAME} completed successfully"
        echo "  Count file: ${COUNT_FILE}"

        # Display the first few lines of the count file for verification
        echo "  First lines of count file:"
        head -n 5 ${COUNT_FILE}
    else
        echo "ERROR: Bowtie2 alignment failed for ${SAMPLE_NAME},
skipping..."
    fi
done
```

```
echo ""
echo "âœ“ All samples processed. Count files in: ${COUNTS_DIR}"
echo ""

# Verify all count files were created
echo ">> Verifying count files:"
ls -lh ${COUNTS_DIR}/*.txt
echo "Total count files: $(ls ${COUNTS_DIR}/*.txt | wc -l)"
echo ""

#
========================================================================
====
# STEP 6: EGGNOG-MAPPER ANNOTATION
#
========================================================================
====
echo
"=======================================================================
========"
echo "STEP 6: FUNCTIONAL ANNOTATION WITH EGGNOG-MAPPER v2.1.12"
echo
"=======================================================================
========"

# Prepare input for eggnog-mapper
EGGNOG_INPUT="${EGGNOG_DIR}/queries.fasta"
EGGNOG_OUTPUT_PREFIX="${EGGNOG_DIR}/out"

# Copy the proteome to eggnog directory
cp ${PROTEOME_FASTA} ${EGGNOG_INPUT}

echo ">> Running EggNOG-mapper v2.1.12..."
echo "   This may take several hours depending on the genome size..."

# Run EggNOG-mapper with diamond algorithm
conda run -n eggnog_env emapper.py \
    --cpu ${THREADS} \
    --mp_start_method forkserver \
    --data_dir /dev/shm/ \
    -o out \
    --output_dir ${EGGNOG_DIR} \
    --temp_dir ${EGGNOG_DIR}/temp \
    --override \
    -m diamond \
    --dmnd_ignore_warnings \
    --dmnd_algo ctg \
    -i ${EGGNOG_INPUT} \
    --evalue 0.001 \
    --score 60 \
    --pident 40 \
    --query_cover 20 \
    --subject_cover 20 \
    --itype proteins \
    --tax_scope 2 \
    --target_orthologs all \
    --go_evidence non-electronic \
    --pfam_realign none \
```

```
    --report_orthologs \
    --decorate_gff yes \
    --excel

# Rename output files to match expected format
if [ -f "${EGGNOG_DIR}/out.emapper.annotations.xlsx" ]; then
    mv ${EGGNOG_DIR}/out.emapper.annotations.xlsx
${EGGNOG_DIR}/EGGNOG_BS253.xlsx
    echo "âœ" Excel output saved to: ${EGGNOG_DIR}/EGGNOG_BS253.xlsx"
fi

if [ -f "${EGGNOG_DIR}/out.emapper.annotations" ]; then
    mv ${EGGNOG_DIR}/out.emapper.annotations
${EGGNOG_DIR}/EGGNOG_BS253.tsv
    echo "âœ" TSV output saved to: ${EGGNOG_DIR}/EGGNOG_BS253.tsv"
fi

# Also decorate the GFF file with EggNOG annotations
if [ -f "${EGGNOG_DIR}/out.emapper.gff" ]; then
    cp ${EGGNOG_DIR}/out.emapper.gff ${GENOME_DIR}/genome_with_eggnog.gff
    echo "âœ" Decorated GFF saved to:
${GENOME_DIR}/genome_with_eggnog.gff"
fi

echo "âœ" EggNOG-mapper completed"
echo ""

# Clean up eggnog temp directory if needed
if [ -d "${EGGNOG_DIR}/temp" ]; then
    rm -rf ${EGGNOG_DIR}/temp
    echo "âœ" Temporary files cleaned up"
fi
```
