# Supplementary material for: Inorganic Carbon Modulates Emulsification Activity and Transcriptional Responses in Vreelandella zhaodongensis BS253
Source: Molecules. 2026 Jun 22;31(12):2182. doi: 10.3390/molecules31122182 (PMC13305022; doi:10.3390/molecules31122182)
Supplement: Supplementary file 1 [file molecules-31-02182-s001.zip › File_S2.R.pdf]

```
#
=========================================================================
====
# INTEGRATED RNA-SEQ PIPELINE
#
=========================================================================
====

# Description:
#   1. Reads count files (*.txt) from the working directory.
#   2. Prepares sample metadata and adds emulsification phenotype.
#   3. Runs DESeq2 and variance stabilizing transformation.
#   4. Generates a styled PCA plot (with COâ,,/NaHCOâ,ƒ subscripts).
#   5. Extracts differentially expressed genes (DEGs) for key contrasts.
#   6. Creates an UpSet plot showing intersections of DEG sets.
#   7. Combines PCA and UpSet into one publicationâ€‘ready figure.
#   8. Performs GO enrichment analysis using a GFF file and
compareCluster.
#   9. Generates a dotplot of enriched GO terms.
#  10. Creates a clean heatmap of transporterâ€‘related DEGs with row
splitting.
#  11. Creates EPS-specific functional heatmap with annotations.
#  12. Integrates emulsification phenotype data and performs Random
Forest.
#  13. Integrates three methods (DESeq2, correlation, Random Forest).
#  14. Generates composite figures for publication.
#
# Requirements:
#   - Count files (*.txt) must be in the working directory.
#   - GFF annotation file (with locus_tag and Ontology_term attributes)
#   - EGGNOG annotation file for EPS analysis
#   - R packages (tidyverse, DESeq2, ComplexHeatmap, clusterProfiler,
etc.)
#
=========================================================================
====

# -----------------------------------------------------------------------
------
# 0. CONFIGURATION â€" ADAPT THESE PATHS AS NEEDED
# -----------------------------------------------------------------------
------
gff_path <- "genome.gff"                    # Path to your GFF annotation
file
eggnog_path <- "EGGNOG_BS253.tsv"           # Path to EGGNOG annotation
file
output_dir <- "Figures_Napp_et_al_finale"    # Main output folder for
figures
count_file_pattern <- "\\.txt$"             # Pattern to identify count
files

# -----------------------------------------------------------------------
------
# 1. SET UP ENVIRONMENT AND INSTALL PACKAGES
# -----------------------------------------------------------------------
------
cat("\n=== 1. SETTING UP ENVIRONMENT AND PACKAGES ===\n")
```

```
if (!requireNamespace("BiocManager", quietly = TRUE))
  install.packages("BiocManager")

bio_pkgs <- c("DESeq2", "ComplexHeatmap", "vsn", "preprocessCore",
              "clusterProfiler", "GO.db", "SummarizedExperiment")
for (p in bio_pkgs) {
  if (!require(p, character.only = TRUE))
    BiocManager::install(p, update = FALSE)
}

cran_pkgs <- c("tidyverse", "pheatmap", "ggrepel", "RColorBrewer",
"grid",
               "patchwork", "ragg", "ggtext", "circlize", "randomForest",
               "UpSetR", "matrixStats")
for (p in cran_pkgs) {
  if (!require(p, character.only = TRUE))
    install.packages(p)
}

# Create output directories
if (!dir.exists(output_dir)) dir.create(output_dir, recursive = TRUE)

# ----------------------------------------------------------------------
------
# 2. IMPORT DATA AND PREPARE COLUMN METADATA (WITH PHENOTYPE INTEGRATION)
# ----------------------------------------------------------------------
------
cat("\n=== 2. IMPORTING DATA AND RENAMING CONDITIONS ===\n")

files <- list.files(".", pattern = count_file_pattern)
if (length(files) == 0) stop("ERROR: No count files found.")

# Extract sample names (assumes format like "C_1_12h_S1.txt")
long_names <- gsub("\\.txt$", "", files)

colData <- data.frame(sample_long = long_names) %>%
  separate(sample_long,
           into = c("id", "cond_raw", "rep", "time", "sid"),
           sep = "_", remove = FALSE) %>%
  mutate(
    time = sub("h", "", time),                # remove "h" suffix
    condition = case_when(
      cond_raw == "C"   ~ "Control",
      cond_raw == "N"   ~ "NaHCO3",
      cond_raw == "CO2" ~ "CO2",
      TRUE ~ cond_raw
    ),
    condition = factor(condition, levels = c("Control", "CO2",
"NaHCO3")),
    group = factor(paste(condition, time, sep = "_"))
  )
rownames(colData) <- paste(colData$condition, colData$rep, colData$time,
sep = "_")

# ----------------------------------------------------------------------
--
# 2.5 INTEGRATE EMULSIFICATION PHENOTYPE DATA
```

```
# ------------------------------------------------------------------------
--
cat("   Integrating emulsification phenotype...\n")

pheno_data <- data.frame(
  condition = rep(c("C", "CO2", "N"), each = 12),
  time      = as.character(rep(c(rep(12, 6), rep(18, 6)), 3)),
  replicate = as.character(rep(rep(1:3, each = 2), 6)),
  value     = c(
    0.000, 3.704, 0.000, 0.000, 3.704, 0.000, 50.000, 3.448, 51.724,
52.632, 3.509, 48.276,
    0.000, 0.000, 0.000, 0.000, 0.000, 0.000, 50.000, 48.276, 50.000,
50.000, 46.667, 44.828,
    51.852, 61.538, 59.259, 62.963, 62.963, 60.714, 63.333, 56.621,
50.000, 61.290, 60.000, 53.333
  )
)

# Map to our sample naming scheme: condition_rep_time
pheno_data$sample <- with(pheno_data, paste0(
  case_when(
    condition == "C" ~ "Control",
    condition == "CO2" ~ "CO2",
    condition == "N" ~ "NaHCO3"
  ),
  "_", replicate, "_", time
))

# Ensure order matches colData
pheno_data <- pheno_data[match(rownames(colData), pheno_data$sample), ]
if (any(is.na(pheno_data$sample))) stop("Phenotype data does not match
sample order!")

# Add emulsification_index to colData
colData$emulsification_index <- pheno_data$value

cat("   Emulsification indices added to colData.\n")
cat("   Summary:\n")
print(summary(colData$emulsification_index))

# ------------------------------------------------------------------------
------
# 3. READ COUNT DATA AND BUILD DESEQ2 OBJECT
# ------------------------------------------------------------------------
------
cat("\n=== 3. READING COUNT DATA ===\n")

cnt_list <- lapply(files, function(f) {
  read.table(f, header = TRUE, comment.char = "#", sep = "\t")[, 7]
})
countData <- do.call(cbind, cnt_list)
rownames(countData) <- read.table(files[1], header = TRUE,
                                  comment.char = "#", sep = "\t")[, 1]
colnames(countData) <- rownames(colData)

cat("   Samples:", paste(colnames(countData), collapse = ", "), "\n")
```

```
# ------------------------------------------------------------------------
------
# 4. DESEQ2 AND VARIANCE STABILIZING TRANSFORMATION
# ------------------------------------------------------------------------
------
cat("\n=== 4. RUNNING DESEQ2 ===\n")

dds <- DESeqDataSetFromMatrix(countData, colData, design = ~ group)
dds <- DESeq(dds)
vsd <- vst(dds, blind = FALSE)

cat("   DESeq2 completed.\n")

# ------------------------------------------------------------------------
------
# 5. FUNCTION TO EXTRACT DEGs FOR A GIVEN CONTRAST
# ------------------------------------------------------------------------
------
cat("\n=== 5. DEFINING DEG EXTRACTION FUNCTION ===\n")

get_deg_list <- function(g1, g2) {
  res <- results(dds, contrast = c("group", g1, g2))
  res_sig <- subset(res, padj < 0.05 & abs(log2FoldChange) > 1)
  return(rownames(res_sig))
}

# ------------------------------------------------------------------------
------
# 6. GENERATE STYLED PCA PLOT (WITH SUBSCRIPTS)
# ------------------------------------------------------------------------
------
cat("\n=== 6. GENERATING PCA PLOT ===\n")

pca_data <- plotPCA(vsd, intgroup = c("condition", "time"), returnData =
TRUE)
percentVar <- round(100 * attr(pca_data, "percentVar"))

# Labels with proper subscripts
pca_labels <- c("Control" = expression(Control),
                "CO2"     = expression(CO[2]),
                "NaHCO3"  = expression(NaHCO[3]))

# Common theme to match reference figures (Bold titles, plain text, clean
axes)
publication_theme <- theme_classic() +
  theme(
    axis.title = element_text(size = 14, face = "bold", color = "black"),
    axis.text  = element_text(size = 12, face = "plain", color =
"black"),
    legend.text = element_text(size = 12, color = "black"),
    legend.title = element_text(size = 11, face = "bold", color =
"black"),
    strip.text = element_text(size = 14, face = "bold", color = "black"),
    plot.title = element_text(size = 14, face = "bold", color = "black",
hjust = 0.5)
  )
```

```
fig_a <- ggplot(pca_data, aes(PC1, PC2, color = condition, shape = time))
+
  geom_point(size = 3) +
  scale_color_manual(
    values = c("Control" = "#333333", "CO2" = "#E41A1C", "NaHCO3" =
"#377EB8"),
    labels = pca_labels
  ) +
  guides(
    color = guide_legend(ncol = 1, order = 1,
                         title.theme = element_text(size = 10, face =
"bold")),
    shape = guide_legend(ncol = 1, order = 2,
                         title.theme = element_text(size = 10, face =
"bold"))
  ) +
  labs(
    x = paste0("PC1: ", percentVar[1], "%"),
    y = paste0("PC2: ", percentVar[2], "%"),
    color = "Condition",
    shape = "Time (h)"
  ) +
  publication_theme +
  theme(
    legend.position = "bottom",
    legend.box = "horizontal",
    legend.direction = "vertical",
    legend.text.align = 0,
    legend.margin = ggplot2::margin(t = 0, r = 0, b = 0, l = 0),
    legend.box.margin = ggplot2::margin(t = -10, r = 0, b = 0, l = 0)
  )

# Top 100 Variable Genes heatmap
# 1. IDENTIFY TOP 100 VARIABLE GENES
# Calculate variance for each gene across all samples
rv <- rowVars(assay(vsd))
select_top100 <- order(rv, decreasing = TRUE)[1:100]

# Subset and scale the matrix (Z-score)
mat_top100 <- assay(vsd)[select_top100, ]
mat_top100_scaled <- t(scale(t(mat_top100)))

# 2. DEFINE ANNOTATIONS (Reusing your established colors)
cond_colors <- c("Control" = "#7FC97F", "CO2" = "#BEAED4", "NaHCO3" =
"#FDC086")
time_colors  <- c("12" = "#333333", "18" = "#CCCCCC")

top_ann_s2 <- HeatmapAnnotation(
  Condition = colData(vsd)$condition,
  Time = as.character(colData(vsd)$time),
  col = list(Condition = cond_colors, Time = time_colors),
  annotation_legend_param = list(
    Condition = list(direction = "horizontal", nrow = 1, title_position =
"topcenter",
                     title_gp = gpar(fontsize = 11, fontface = "bold"),
labels_gp = gpar(fontsize = 10)),
    Time = list(direction = "horizontal", nrow = 1, title_position =
"topcenter",
```

```
                title_gp = gpar(fontsize = 11, fontface = "bold"),
labels_gp = gpar(fontsize = 10))
  )
)

# 3. CONSTRUCT HEATMAP
h_s2 <- Heatmap(
  mat_top100_scaled,
  name = "Z-score",
  col = colorRamp2(c(-2, 0, 2), c("#2166ac", "white", "#b2182b")),

  # Clustering logic
  cluster_rows = TRUE,
  cluster_columns = TRUE,
  show_row_dend = TRUE,
  show_column_dend = TRUE,

  # Visuals
  show_row_names = FALSE, # 100 names usually clutter a supplementary
figure
  show_column_names = FALSE,
  top_annotation = top_ann_s2,

  column_title = "Figure S2: Top 100 Variable Genes",
  column_title_gp = gpar(fontsize = 12, fontface = "bold"),

  heatmap_legend_param = list(
    direction = "horizontal",
    title_position = "topcenter",
    title_gp = gpar(fontsize = 11, fontface = "bold"),
    labels_gp = gpar(fontsize = 10),
    legend_width = unit(4, "cm")
  )
)

# 4. EXPORT AS HIGH-RES TIFF
output_s2 <- "Figures_Napp_et_al/Figure_S2.tiff"

while (length(dev.list()) > 0) dev.off()

agg_tiff(output_s2, width = 210, height = 260, units = "mm",
         res = 600, compression = "lzw")

# Apply the centralized legend alignment you preferred
draw(h_s2,
     heatmap_legend_side = "bottom",
     annotation_legend_side = "bottom",
     merge_legends = TRUE,
     padding = unit(c(5, 5, 20, 5), "mm"))

dev.off()

cat("âœ" Figure S2 (Top 100 Variable Genes) saved to:", output_s2, "\n")


# ------------------------------------------------------------
------
# 7. EXTRACT DEGs FOR UPSET PLOT
```

```
# -----------------------------------------------------------------------
------
cat("\n=== 7. EXTRACTING DEG SETS ===\n")

upset_list <- list(
  "Control"              = get_deg_list("Control_18", "Control_12"),
  "CO2"                  = get_deg_list("CO2_18", "CO2_12"),
  "NaHCO3"               = get_deg_list("NaHCO3_18", "NaHCO3_12"),
  "NaHCO3_vs_Control_18" = get_deg_list("NaHCO3_18", "Control_18"),
  "CO2_vs_Control_18"    = get_deg_list("CO2_18", "Control_18")
)

# Remove empty sets
upset_list <- upset_list[sapply(upset_list, length) > 0]

if (length(upset_list) >= 2) {
  # ---------------------------------------------------------------------
------
  # 8. GENERATE UPSET PLOT (ComplexHeatmap) AND COMBINED FIGURE
  # ---------------------------------------------------------------------
------
  cat("\n=== 8. GENERATING UPSET PLOT AND COMPOSITE FIGURE ===\n")

  m <- make_comb_mat(upset_list)

  # Row labels with subscripts
  row_labels_list <- list(
    "Control"              = bquote("Control (18 vs 12 h)"),
    "CO2"                  = bquote(CO[2]~"(18 vs 12 h)"),
    "NaHCO3"               = bquote(NaHCO[3]~"(18 vs 12 h)"),
    "NaHCO3_vs_Control_18" = bquote(NaHCO[3]~"vs Control (18 h)"),
    "CO2_vs_Control_18"    = bquote(CO[2]~"vs Control (18 h)")
  )
  final_row_labels <-
as.expression(unname(row_labels_list[names(upset_list)]))

  ht <- UpSet(
    m,
    set_order = names(upset_list),
    comb_order = order(comb_size(m), decreasing = TRUE),
    row_labels = final_row_labels,
    pt_size = unit(1.5, "mm"),
    lwd = 1.0,
    row_names_gp = grid::gpar(fontsize = 10),
    column_names_gp = grid::gpar(fontsize = 10),
    top_annotation = upset_top_annotation(
      m,
      add_numbers = TRUE,
      numbers_gp = grid::gpar(fontsize = 8, fontface = "plain"),
      numbers_rot = 60,
      height = unit(6, "cm"),
      annotation_name_gp = grid::gpar(fontsize = 10, fontface = "bold")
    ),
    height = unit(1.8, "cm"),
    right_annotation = upset_right_annotation(
      m,
      add_numbers = TRUE,
      numbers_gp = grid::gpar(fontsize = 8, fontface = "plain"),
```

```
      annotation_name_gp = grid::gpar(fontsize = 10, fontface = "bold")
    )
  )

    # Convert to grob and combine with PCA
  fig_b_grob <- grid::grid.grabExpr(draw(ht))

  combined_plot <- (fig_a | fig_b_grob) +
    plot_layout(widths = c(0.5, 1.5)) +
    plot_annotation(tag_levels = "A") &
    theme(plot.tag = element_text(size = 18, face = "bold"))

  out_file <- file.path(output_dir, "Figure_5.tiff")
  agg_tiff(out_file,
           width = 175, height = 125, units = "mm",
           res = 600, compression = "lzw")
  print(combined_plot)
  dev.off()
  cat("   Composite figure saved to:", out_file, "\n")
}

# -----------------------------------------------------------------------
------
# 9. FUNCTIONAL ENRICHMENT FROM GFF FILE
# -----------------------------------------------------------------------
------
cat("\n=== 9. EXTRACTING GO TERMS FROM GFF AND RUNNING ENRICHMENT ===\n")

if (!file.exists(gff_path)) stop("GFF file not found: ", gff_path)

# ---- 9.1. Read GFF and extract GO terms ----
gff_raw <- read_delim(gff_path, delim = "\t", comment = "#", col_names =
FALSE,
                      show_col_types = FALSE)

t2g <- gff_raw %>%
  dplyr::filter(X3 == "CDS") %>%
  dplyr::mutate(
    Gene = stringr::str_extract(X9, "(?<=locus_tag=)[^;]+"),
    Terms = stringr::str_extract(X9, "(?<=Ontology_term=)[^;]+")
  ) %>%
  dplyr::filter(!is.na(Terms)) %>%
  tidyr::separate_rows(Terms, sep = ",") %>%
  dplyr::select(Term = Terms, Gene = Gene) %>%
  dplyr::distinct()

# Fallback method if read_delim failed
if (nrow(t2g) == 0) {
  message("   No GO terms extracted with read_delim. Trying readLines
fallback...")
  lines <- readLines(gff_path)
  cds_lines <- lines[grepl("\tCDS\t", lines) & grepl("Ontology_term=",
lines)]

  t2g <- data.frame(raw = cds_lines) %>%
    dplyr::mutate(
      Gene = stringr::str_extract(raw, "(?<=locus_tag=)[^;]+"),
      Terms = stringr::str_extract(raw, "(?<=Ontology_term=)[^;]+")
```

```
    ) %>%
    tidyr::separate_rows(Terms, sep = ",") %>%
    dplyr::select(Term = Terms, Gene = Gene) %>%
    dplyr::distinct()
}

cat("   Associations found:", nrow(t2g), "\n")

# ---- 9.2. Map GO IDs to names ----
all_go_ids <- unique(t2g$Term)
t2n <- data.frame(
  Term = all_go_ids,
  Name = sapply(all_go_ids, function(x) {
    term_info <- GOTERM[[x]]
    if (!is.null(term_info)) return(Term(term_info)) else return(x)
  })
)

# ---- 9.3. Run compareCluster ----
enrich <- compareCluster(geneClusters = upset_list,
                         fun = "enricher",
                         TERM2GENE = t2g,
                         TERM2NAME = t2n)

# ---- 9.4. Format cluster labels with subscripts ----
df_enrich <- as.data.frame(enrich)

cluster_labels <- c(
  "Control"              = "Control (18 vs 12 h)",
  "CO2"                  = "CO<sub>2</sub> (18 vs 12 h)",
  "NaHCO3"               = "NaHCO<sub>3</sub> (18 vs 12 h)",
  "NaHCO3_vs_Control_18" = "NaHCO<sub>3</sub> vs C (18 h)",
  "CO2_vs_Control_18"    = "CO<sub>2</sub> vs C (18 h)"
)

present_clusters <- unique(df_enrich$Cluster)
df_enrich$Cluster <- cluster_labels[as.character(df_enrich$Cluster)]
df_enrich$Cluster <- factor(df_enrich$Cluster,
                            levels =
unname(cluster_labels[names(cluster_labels) %in% present_clusters]))

enrich@compareClusterResult <- df_enrich

# ---- 9.5. Generate dotplot ----
p_enrich <- dotplot(enrich, showCategory = 10) +
  scale_y_discrete(labels = function(x) str_wrap(x, width = 45)) +
  publication_theme +
  theme(
    axis.text.x = element_markdown(angle = 60, hjust = 1, size = 10,
                                   face = "bold", color = "black"),
    panel.grid.major = element_line(linewidth = 0.2, color = "grey90")
  ) +
  labs(x = NULL, y = "Enriched GO Terms")

# -----------------------------------------------------------------
------
# 10. CLEAN TRANSPORTER HEATMAP â€" WITH SHORT LABELS (NO ROTATION)
```

```
# ---------------------------------------------------------------------------
------
cat("\n=== 10. GENERATING TRANSPORTER HEATMAP ===\n")

terms_of_interest <- c(
  "ATPase-coupled transmembrane transporter activity",
  "ABC-type transporter activity",
  "carbohydrate transport"
)

# ---- 10.1. Filter and map genes ----
gene_func_map_clean <- t2g %>%
  inner_join(t2n, by = "Term", relationship = "many-to-many") %>%
  dplyr::filter(Name %in% terms_of_interest) %>%
  distinct(Gene, Name) %>%
  mutate(Name = factor(Name, levels = terms_of_interest)) %>%
  arrange(Gene, Name) %>%
  group_by(Gene) %>%
  summarize(Function = dplyr::first(Name)) %>%
  ungroup()

all_degs <- unique(unlist(upset_list))
genes_to_plot <- intersect(gene_func_map_clean$Gene, all_degs)

# Define the updated terms with the line break for the plot
plot_terms <- str_replace(terms_of_interest,
                          "ATPase-coupled transmembrane transporter
activity",
                          "ATPase-coupled transmembrane\ntransporter
activity")

gene_func_map_clean <- gene_func_map_clean %>%
  dplyr::filter(Gene %in% genes_to_plot) %>%
  mutate(Function = factor(str_replace(as.character(Function),
                                       "ATPase-coupled transmembrane
transporter activity",
                                       "ATPase-coupled
transmembrane\ntransporter activity"),
                          levels = plot_terms))

if (length(genes_to_plot) > 0) {
  # ---- 10.2. Zâ€‘score matrix ----
  mat_scaled <- t(scale(t(assay(vsd)[genes_to_plot, ])))

  # Remove any rows that became NA after scaling (e.g., zero variance)
  valid_rows <- complete.cases(mat_scaled)
  if (!all(valid_rows)) {
    cat("   Removing", sum(!valid_rows), "rows with NA after scaling.\n")
    mat_scaled <- mat_scaled[valid_rows, , drop = FALSE]
    genes_to_plot <- genes_to_plot[valid_rows]
    gene_func_map_clean <- gene_func_map_clean %>% dplyr::filter(Gene
%in% genes_to_plot)
  }

  if (nrow(mat_scaled) > 0) {
    # ---- 10.3. Top annotations (Condition and Time) ----
    col_names <- colnames(mat_scaled)
    cond_vec <- case_when(
```

```
      str_detect(col_names, "Control") ~ "Control",
      str_detect(col_names, "CO2")      ~ "CO2",
      str_detect(col_names, "NaHCO3")   ~ "NaHCO3",
      TRUE ~ "Other"
    )
    time_vec <- case_when(
      str_detect(col_names, "12") ~ "12h",
      str_detect(col_names, "18") ~ "18h",
      TRUE ~ "Unknown"
    )

    cond_colors <- c("Control" = "#7FC97F", "CO2" = "#BEAED4", "NaHCO3" =
"#FDC086")
    time_colors <- c("12h" = "#333333", "18h" = "#CCCCCC")

    top_ann <- HeatmapAnnotation(
      Condition = cond_vec,
      Time = time_vec,
      col = list(Condition = cond_colors, Time = time_colors),
      simple_anno_size = unit(3.5, "mm"),
      annotation_name_side = "right",
      annotation_name_gp = gpar(fontsize = 10, fontface = "bold"),
      annotation_legend_param = list(
        Condition = list(title_gp = gpar(fontsize = 11, fontface =
"bold"), labels_gp = gpar(fontsize = 10)),
        Time = list(title_gp = gpar(fontsize = 11, fontface = "bold"),
labels_gp = gpar(fontsize = 10))
      )
    )

    # ---- 10.4. Left annotation (Function + short DEG labels) ----
    deg_presence <- sapply(upset_list, function(x)
as.numeric(genes_to_plot %in% x))

    # Short labels that fit without rotation
    deg_col_labels <- c(
      "Control"              = "C 18v12",
      "CO2"                  = "CO\u2082 18v12",
      "NaHCO3"               = "Na 18v12",
      "NaHCO3_vs_Control_18" = "Na v C(18)",
      "CO2_vs_Control_18"    = "CO\u2082 v C(18)"
    )
    colnames(deg_presence) <- deg_col_labels[names(upset_list)]

    col_fun <- colorRamp2(c(-2, 0, 2), c("#377EB8", "white", "#E41A1C"))
    deg_col_fun <- c("0" = "white", "1" = "#4DAF4A")
    func_colors <- setNames(c("#8DD3C7", "#BEBADA", "#FB8072"),
plot_terms)


    # Create left annotation
    left_ann <- HeatmapAnnotation(
      which = "row",
      Function = gene_func_map_clean$Function,
      `Is DEG` = deg_presence,
      col = list(
        Function = func_colors,
        `Is DEG` = deg_col_fun
```

```
    ),
    show_legend = c(FALSE, FALSE),
    simple_anno_size = unit(3, "mm"),
    annotation_name_side = "top",
    annotation_name_gp = gpar(fontsize = 10, fontface = "bold")
  )

  # ---- 10.5. Heatmap parameters ----
  h_plot <- Heatmap(
    mat_scaled,
    name = "Z-score",
    col = col_fun,
    top_annotation = top_ann,
    left_annotation = left_ann,
    row_split = gene_func_map_clean$Function,
    row_title_rot = 0,
    row_title_gp = gpar(fontsize = 10, fontface = "bold"),
    show_column_names = FALSE,
    width = unit(100, "mm"),
    row_names_gp = gpar(fontsize = 5),
    row_names_max_width = unit(12, "cm"),
    cluster_columns = TRUE,
    show_row_dend = TRUE,
    heatmap_legend_param = list(
      direction = "horizontal",
      title_position = "topcenter",
      title_gp = gpar(fontsize = 11, fontface = "bold"),
      labels_gp = gpar(fontsize = 10)
    )
  )

  # ------------------------------------------------------------------------
------
  # 11. COMBINE FIGURE 5 AND FIGURE 6 SIDE BY SIDE (A | B)
  # ------------------------------------------------------------------------
------
  cat("\n=== 11. CREATING COMPOSITE FIGURE (ENRICHMENT + HEATMAP)
===\n")

  # Convert heatmap to grob
  fig_B_grob <- grid::grid.grabExpr(
    draw(h_plot,
         heatmap_legend_side = "bottom",
         annotation_legend_side = "bottom",
         merge_legends = TRUE,
         padding = unit(c(5, 20, 5, 10), "mm"))
  )

  # Combine with enrichment plot
  fig_combined_AB <- (p_enrich | fig_B_grob) +
    plot_layout(widths = c(0.4, 1.7)) +
    plot_annotation(tag_levels = "A") &
    theme(plot.tag = element_text(size = 26, face = "bold"))

  # Save composite figure
  composite_file <- file.path(output_dir, "Figure_6.tiff")
  agg_tiff(composite_file,
           width = 400, height = 200, units = "mm",
```

```
            res = 600, compression = "lzw")
    print(fig_combined_AB)
    dev.off()

    cat("   Composite figure (enrichment + heatmap) saved to:",
composite_file, "\n")
  }
}

# --------------------------------------------------------------------------
------
# 12. EPS-SPECIFIC FUNCTIONAL HEATMAP
# --------------------------------------------------------------------------
------
cat("\n=== 12. GENERATING EPS-SPECIFIC FUNCTIONAL HEATMAP ===\n")

if (file.exists(eggnog_path)) {
  # ---- 12.1. Load and filter EGGNOG annotations ----
  eggnog_raw <- read.table(eggnog_path, sep = "\t", header = FALSE,
                           comment.char = "#", quote = "", fill = TRUE)

  colnames(eggnog_raw) <- c("query", "seed_ortholog", "evalue", "score",
"eggNOG_OGs",
                            "max_annot_lvl", "COG_category",
"Description", "Preferred_name",
                            "GOs", "EC", "KEGG_ko", "KEGG_Pathway",
"KEGG_Module",
                            "KEGG_Reaction", "KEGG_rclass", "BRITE",
"KEGG_TC", "CAZy",
                            "BiGG_Reaction", "PFAMs")

  # Filter for EPS-related proteins
  eps_targets <- eggnog_raw %>%
    filter(str_detect(COG_category, "M") |
             str_detect(Description,
"(?i)polysaccharide|glycosyltransferase|flippase|polymerase|sugar-
nucleotide|wzx|wzy|wza"))

  # ---- 12.2. Map protein IDs to locus tags ----
  gff_map <- gff_raw %>%
    filter(X3 == "CDS") %>%
    mutate(
      protein_id = str_extract(X9, "(?<=cds-)[^;]+"),
      locus_tag = str_extract(X9, "(?<=gene-)[^;]+")
    ) %>%
    filter(!is.na(protein_id) & !is.na(locus_tag)) %>%
    dplyr::select(protein_id, locus_tag) %>%
    distinct()

  eps_final_list <- eps_targets %>%
    left_join(gff_map, by = c("query" = "protein_id")) %>%
    filter(!is.na(locus_tag))

  # ---- 12.3. Filter DEGs & prepare matrix ----
  all_degs <- unique(unlist(upset_list))
  eps_deg_final <- eps_final_list %>%
    filter(locus_tag %in% all_degs) %>%
    filter(locus_tag %in% rownames(vsd))
```

```
  # ---- 12.4. Save EPS annotation CSV ----
  if (nrow(eps_deg_final) > 0) {

    # Calculate correlations for each EPS gene
    eps_genes <- eps_deg_final$locus_tag
    expr_matrix_eps <- assay(vsd)[eps_genes, , drop = FALSE]
    correlations <- apply(expr_matrix_eps, 1, function(x) cor(x,
colData$emulsification_index))

    # Create annotation table for EPS genes
    eps_annotation_table <- eps_deg_final %>%
      dplyr::select(
        Gene_ID = locus_tag,
        Protein_ID = query,
        Description = Description,
        Preferred_Name = Preferred_name,
        COG_Category = COG_category,
        EC_Number = EC,
        KEGG_KO = KEGG_ko,
        KEGG_Pathway = KEGG_Pathway,
        CAZy = CAZy,
        PFAMs = PFAMs
      ) %>%
      # Add mapping source
      mutate(
        Mapping_Source = case_when(
          str_detect(COG_Category, "M") & str_detect(Description,
"(?i)polysaccharide|glycosyltransferase|flippase|polymerase|sugar-
nucleotide|wzx|wzy|wza") ~ "COG_M + Keyword",
          str_detect(COG_Category, "M") ~ "COG_Category_M",
          str_detect(Description,
"(?i)polysaccharide|glycosyltransferase|flippase|polymerase|sugar-
nucleotide|wzx|wzy|wza") ~ "Keyword_Match",
          TRUE ~ "Other"
        ),
        # Add expression correlation with emulsification
        Correlation_with_Emulsification = correlations[Gene_ID]
      ) %>%
      # Add DEG status in each comparison
      mutate(
        in_Control_18vs12 = Gene_ID %in% upset_list$Control,
        in_CO2_18vs12 = Gene_ID %in% upset_list$CO2,
        in_NaHCO3_18vs12 = Gene_ID %in% upset_list$NaHCO3,
        in_NaHCO3_vs_Control_18 = Gene_ID %in%
upset_list$NaHCO3_vs_Control_18,
        in_CO2_vs_Control_18 = Gene_ID %in% upset_list$CO2_vs_Control_18
      ) %>%
      arrange(desc(abs(Correlation_with_Emulsification)))

    # Save CSV
    csv_path <- file.path(output_dir, "Table_S1.csv")
    write.csv(eps_annotation_table, csv_path, row.names = FALSE)
    cat(sprintf("   âœ" EPS annotation table saved to: %s (%d genes)\n",
csv_path, nrow(eps_annotation_table)))

    # ---- 12.5. Continue with heatmap ----
    valid_genes <- eps_deg_final$locus_tag
```

```
    pheno_aligned <- pheno_data %>% filter(sample %in% colnames(vsd)) %>%
      arrange(match(sample, colnames(vsd)))
    ei_values <- pheno_aligned$value
    exp_mat <- assay(vsd)[valid_genes, ]
    cors <- apply(exp_mat, 1, function(x) cor(x, ei_values, method =
"pearson"))
    genes_sorted <- names(sort(cors, decreasing = TRUE))

    # ---- 12.6. Categorization ----
    eps_deg_final <- eps_deg_final %>%
      mutate(Category = case_when(
        str_detect(Description, "(?i)glycosyltransferase") ~
"Glycosyltransferase",
        str_detect(Description, "(?i)flippase|wzx|wzy|polymerase") ~
"Export/Polymerization",
        str_detect(Description, "(?i)sugar-nucleotide|ugd|galU|glmS") ~
"Precursor Metabolism",
        str_detect(COG_category, "M") ~ "Cell Wall/Envelope",
        TRUE ~ "Other EPS-related"
      ))

    mat_eps_scaled <- t(scale(t(exp_mat[genes_sorted, ])))
    row_labs_vec <- eps_deg_final %>%
      filter(locus_tag %in% genes_sorted) %>%
      mutate(display_name = ifelse(Preferred_name == "-" |
is.na(Preferred_name) | Preferred_name == "",
                                   locus_tag, paste0(Preferred_name, "
(", locus_tag, ")"))) %>%
      distinct(locus_tag, .keep_all = TRUE) %>%
      arrange(match(locus_tag, genes_sorted)) %>%
      pull(display_name)

    cat_vector <- eps_deg_final %>%
      filter(locus_tag %in% genes_sorted) %>%
      arrange(match(locus_tag, genes_sorted)) %>%
      pull(Category)

    # ---- 12.7. Colors & annotations ----
    cond_colors <- c("Control" = "#7FC97F", "CO2" = "#BEAED4", "NaHCO3" =
"#FDC086")
    time_colors  <- c("12" = "#333333", "18" = "#CCCCCC")
    deg_col_fun  <- c("0" = "white", "1" = "#4DAF4A")
    cat_colors <- c("Glycosyltransferase" = "#E41A1C",
                    "Export/Polymerization" = "#377EB8",
                    "Precursor Metabolism" = "#984EA3",
                    "Cell Wall/Envelope" = "#FF7F00",
                    "Other EPS-related" = "#A65628")

    # Prepare annotations
    deg_presence <- sapply(upset_list, function(x)
as.numeric(genes_sorted %in% x))
    colnames(deg_presence) <- c("C 18v12", "CO\u2082 18v12", "Na 18v12",
"Na v C(18)", "CO\u2082 v C(18)")

    left_ann <- rowAnnotation(
      `Is DEG` = deg_presence,
      col = list(`Is DEG` = deg_col_fun),
      show_legend = FALSE,
```

```
      simple_anno_size = unit(3, "mm"),
      annotation_name_side = "top",
      annotation_name_gp = gpar(fontsize = 10, fontface = "bold")
    )

    top_ann <- HeatmapAnnotation(
      Condition = colData(vsd)$condition,
      Time = colData(vsd)$time,
      col = list(Condition = cond_colors, Time = time_colors),
      annotation_legend_param = list(
        Condition = list(direction = "horizontal", nrow = 1,
title_position = "topcenter",
                         title_gp = gpar(fontsize = 11, fontface =
"bold"), labels_gp = gpar(fontsize = 10)),
        Time = list(direction = "horizontal", nrow = 1, title_position =
"topcenter",
                    title_gp = gpar(fontsize = 11, fontface = "bold"),
labels_gp = gpar(fontsize = 10))
      )
    )

    right_ann <- rowAnnotation(
      Gene = anno_text(row_labs_vec, gp = gpar(fontsize = 6)),
      Function = cat_vector,
      col = list(Function = cat_colors),
      annotation_legend_param = list(
        Function = list(direction = "horizontal", nrow = 1,
title_position = "topcenter",
                        title_gp = gpar(fontsize = 11, fontface =
"bold"), labels_gp = gpar(fontsize = 10))
      )
    )

    # ---- 12.8. Heatmap construction ----
    h_final <- Heatmap(
      mat_eps_scaled,
      name = "Z-score",
      col = colorRamp2(c(-2, 0, 2), c("#2166ac", "white", "#b2182b")),
      column_order = order(factor(colData(vsd)$condition, levels =
c("Control", "CO2", "NaHCO3")),
                           factor(colData(vsd)$time, levels = c("12",
"18"))),
      cluster_rows = FALSE,
      show_row_names = FALSE,
      show_column_names = FALSE,
      top_annotation = top_ann,
      left_annotation = left_ann,
      right_annotation = right_ann,
      heatmap_legend_param = list(
        direction = "horizontal",
        title_position = "topcenter",
        title_gp = gpar(fontsize = 11, fontface = "bold"),
        labels_gp = gpar(fontsize = 10)
      )
    )

    # ---- 12.9. TIFF export ----
    output_path <- file.path(output_dir, "Figure_7.tiff")
```

```
    agg_tiff(output_path, width = 230, height = 300, units = "mm",
             res = 600, compression = "lzw")

    draw(h_final,
         heatmap_legend_side = "bottom",
         annotation_legend_side = "bottom",
         merge_legends = TRUE,
         padding = unit(c(5, 5, 25, 45), "mm"))

    dev.off()
    cat("   EPS heatmap saved to:", output_path, "\n")

    # Print summary
    cat("\n--- EPS Gene Summary ---\n")
    cat(sprintf("Total EPS-related genes: %d\n",
nrow(eps_annotation_table)))
    cat(sprintf("  - Glycosyltransferases: %d\n",
sum(eps_deg_final$Category == "Glycosyltransferase")))
    cat(sprintf("  - Export/Polymerization: %d\n",
sum(eps_deg_final$Category == "Export/Polymerization")))
    cat(sprintf("  - Precursor Metabolism: %d\n",
sum(eps_deg_final$Category == "Precursor Metabolism")))
    cat(sprintf("  - Cell Wall/Envelope: %d\n",
sum(eps_deg_final$Category == "Cell Wall/Envelope")))
    cat(sprintf("  - Other EPS-related: %d\n", sum(eps_deg_final$Category
== "Other EPS-related")))

  } else {
    cat("   No EPS-related DEGs found.\n")
  }
} else {
  cat("   EGGNOG file not found. Skipping EPS analysis.\n")
}

#
========================================================================
====
# ML INTEGRATION: RANDOM FOREST + CORRECTED CORRELATION ANALYSIS
#
========================================================================
====
cat("\n=== ML INTEGRATION: ADJUSTED RANDOM FOREST AND CORRELATION ===\n")

# Get all unique genes from upset_list
all_genes <- unique(unlist(upset_list))
cat(sprintf("   Total unique DEGs across all contrasts: %d\n",
length(all_genes)))

if (length(all_genes) == 0) {
  stop("ERROR: No DEGs found. Cannot proceed with ML analysis.")
}

# ------------------------------------------------------------------------
------
# 1. CALCULATE EVIDENCE COUNTS (UNCHANGED)
# ------------------------------------------------------------------------
------
cat("\n--- Step 1: Calculating evidence counts ---\n")
```

```
evidence_counts <- rep(0, length(all_genes))
names(evidence_counts) <- all_genes

for (contrast_name in names(upset_list)) {
  genes_in_contrast <- upset_list[[contrast_name]]
  evidence_counts[genes_in_contrast] <-
evidence_counts[genes_in_contrast] + 1
}

print(table(evidence_counts))

# ---------------------------------------------------------------------------
------
# 2. ADJUSTED CORRELATION (REMOVE CONDITION + TIME EFFECTS)
# ---------------------------------------------------------------------------
------
cat("\n--- Step 2: Calculating ADJUSTED correlations ---\n")

expr_matrix <- assay(vsd)[all_genes, , drop = FALSE]

# Residualize phenotype
pheno_res <- resid(lm(emulsification_index ~ condition + time, data =
colData))

# Residualize expression
expr_res <- t(apply(expr_matrix, 1, function(x) {
  resid(lm(x ~ colData$condition + colData$time))
}))

# Correlate residuals
correlations <- apply(expr_res, 1, function(x) {
  cor(x, pheno_res, method = "pearson", use = "complete.obs")
})

cat(sprintf("   Adjusted correlation range: %.3f to %.3f\n",
            min(correlations, na.rm = TRUE),
            max(correlations, na.rm = TRUE)))

cat(sprintf("   Genes with |correlation| > 0.5: %d\n",
            sum(abs(correlations) > 0.5, na.rm = TRUE)))

# ---------------------------------------------------------------------------
------
# 3. FEATURE SELECTION (ROBUST)
# ---------------------------------------------------------------------------
------
cat("\n--- Step 3: Feature selection for RF ---\n")

# Combine evidence + correlation
candidate_pool <- data.frame(
  gene = all_genes,
  correlation = correlations,
  evidence = evidence_counts
)

candidate_pool <- candidate_pool %>%
  dplyr::filter(evidence >= 2) %>%
```

```r
  dplyr::arrange(desc(abs(correlation)))

# Limit features based on sample size
n_samples <- ncol(expr_res)
max_features <- min(30, floor(n_samples * 0.8), nrow(candidate_pool))

top_genes <- candidate_pool$gene[1:max_features]

cat(sprintf("   Using %d genes for RF\n", length(top_genes)))

# ------------------------------------------------------------------------
------
# 4. RANDOM FOREST (ON RESIDUALS)
# ------------------------------------------------------------------------
------
cat("\n--- Step 4: Running Random Forest (adjusted data) ---\n")

rf_data <- as.data.frame(t(expr_res[top_genes, , drop = FALSE]))
rf_data$emulsification <- pheno_res

if (ncol(rf_data) > 2 && ncol(rf_data) <= nrow(rf_data)) {

  set.seed(42)

  mtry_values <- seq(2, min(10, floor(sqrt(ncol(rf_data) - 1))), by = 1)
  if (length(mtry_values) == 0) mtry_values <- 2

  oob_errors <- c()
  for (mtry_val in mtry_values) {
    rf_tune <- randomForest(
      emulsification ~ .,
      data = rf_data,
      ntree = 1000,
      mtry = mtry_val
    )
    oob_errors <- c(oob_errors, tail(rf_tune$mse, 1))
  }

  best_mtry <- mtry_values[which.min(oob_errors)]
  cat(sprintf("   Best mtry: %d\n", best_mtry))

  rf_model <- randomForest(
    emulsification ~ .,
    data = rf_data,
    importance = TRUE,
    ntree = 500,
    mtry = best_mtry
  )

  rf_importance <- importance(rf_model)

  rf_importance_df <- data.frame(
    gene = rownames(rf_importance),
    IncMSE = rf_importance[, "%IncMSE"],
    stringsAsFactors = FALSE
  ) %>%
    dplyr::arrange(desc(IncMSE))
```

```
  cat(sprintf("   Adjusted RF completed. RÂ²: %.3f\n", tail(rf_model$rsq,
1)))
  print(head(rf_importance_df, 10))

} else {
  cat("   WARNING: RF not feasible\n")
  rf_importance_df <- data.frame(
    gene = top_genes,
    IncMSE = NA
  )
  rf_model <- NULL
}

# ----------------------------------------------------------------------------
------
# 5. CREATE FINAL CANDIDATE TABLE (UPDATED WITH DESCRIPTIONS)
# ----------------------------------------------------------------------------
------
cat("\n--- Step 5: Creating candidate table ---\n")

# 1. Prepare annotation mapping (Locus Tag -> Description & Name)
# We map the full EGGNOG dataset (not just EPS genes) to get descriptions
for all DEGs
gene_annotations <- eggnog_raw %>%
  left_join(gff_map, by = c("query" = "protein_id")) %>%
  dplyr::filter(!is.na(locus_tag)) %>%
  dplyr::select(locus_tag, Preferred_name, Description) %>%
  dplyr::distinct(locus_tag, .keep_all = TRUE)


# 2. Build the base candidate table
candidate_table <- data.frame(
  gene = all_genes,
  correlation = correlations,
  evidence_count = evidence_counts
)

# 3. Add Random Forest Importance
candidate_table$RF_IncMSE <- rf_importance_df$IncMSE[
  match(candidate_table$gene, rf_importance_df$gene)
]

# 4. Calculate Ranks and Join Annotations
candidate_table <- candidate_table %>%
  mutate(
    correlation_rank = rank(-abs(correlation), na.last = "keep"),
    evidence_rank = rank(-evidence_count),
    rf_rank = rank(-RF_IncMSE, na.last = "keep"),
    combined_score = (correlation_rank + evidence_rank + rf_rank) / 3
  ) %>%
  # JOIN DESCRIPTIONS HERE
  left_join(gene_annotations, by = c("gene" = "locus_tag")) %>%
  # Organize columns so name and description are at the front
  dplyr::select(gene, Preferred_name, Description, everything()) %>%
  arrange(combined_score)

# 5. Save the final Table S2
write.csv(candidate_table,
```

```
          file.path(output_dir, "Table_S2.csv"),
          row.names = FALSE)

cat("    âœ" Adjusted candidate table with gene descriptions saved as
Table_S2.csv\n")


# -------------------------------------------------------------------------
------
# 1. PREPARE DATA
# -------------------------------------------------------------------------
------

plot_df <- candidate_pool %>%
  dplyr::left_join(rf_importance_df, by = "gene") %>%
  dplyr::filter(!is.na(IncMSE))

plot_df$direction <- ifelse(plot_df$correlation > 0, "Positive",
"Negative")

# -------------------------------------------------------------------------
------
# 2. PANEL A: MULTI-DIMENSIONAL SCATTER
# -------------------------------------------------------------------------
------

pA <- ggplot(plot_df, aes(x = correlation, y = IncMSE)) +
  geom_point(aes(size = evidence, color = direction), alpha = 0.8) +

  geom_text_repel(
    data = plot_df %>% dplyr::slice_max(order_by = IncMSE, n = 10),
    aes(label = gene),
    size = 3.5,
    max.overlaps = Inf
  ) +

  scale_color_manual(values = c("Positive" = "#D55E00", "Negative" =
"#0072B2")) +

  labs(
    x = "Adjusted Correlation",
    y = "%IncMSE",
    size = "Evidence",
    color = "Direction"
  ) +

  publication_theme

# -------------------------------------------------------------------------
------
# 3. PANEL B: TOP GENES BARPLOT
# -------------------------------------------------------------------------
------

top_plot <- plot_df %>%
  dplyr::arrange(desc(IncMSE)) %>%
  dplyr::slice(1:10)
```

```
pB <- ggplot(top_plot, aes(x = reorder(gene, IncMSE), y = IncMSE)) +
  geom_bar(stat = "identity", aes(fill = correlation)) +
  coord_flip() +

  scale_fill_gradient2(
    low = "#0072B2",
    mid = "white",
    high = "#D55E00",
    midpoint = 0
  ) +

  labs(
    x = "Gene",
    y = "%IncMSE",
    fill = "Correlation"
  ) +

  publication_theme

# -------------------------------------------------------------------------
------
# 4. PANEL C: CORRELATION DISTRIBUTION BY EVIDENCE
# -------------------------------------------------------------------------
------

pC <- ggplot(candidate_pool, aes(x = correlation, fill =
factor(evidence))) +
  geom_density(alpha = 0.4) +

  labs(
    x = "Adjusted Correlation",
    fill = "Evidence"
  ) +

  publication_theme


# -------------------------------------------------------------------------
------
# 5. PANEL D: CORRELATION vs RF AGREEMENT
# -------------------------------------------------------------------------
------

pD <- ggplot(plot_df, aes(x = abs(correlation), y = IncMSE)) +
  geom_point(aes(color = evidence), size = 3, alpha = 0.8) +

  geom_smooth(method = "lm", se = FALSE, linetype = "dashed") +

  labs(
    x = "|Correlation|",
    y = "%IncMSE",
    color = "Evidence"
  ) +

  publication_theme

# -------------------------------------------------------------------------
------
```

```
# 6. COMBINE PANELS (PATCHWORK)
# ----------------------------------------------------------------------
------

final_plot <- (pA | pB) / (pC | pD) +
  plot_annotation(tag_levels = "A") &
  theme(plot.tag = element_text(size = 24, face = "bold"))

# ----------------------------------------------------------------------
------
# SAVE AS HIGH-QUALITY TIFF
# ----------------------------------------------------------------------
------
ggsave(
  filename = "Figures_Napp_et_al_finale/Figure_8.tiff",
  plot = final_plot,
  device = "tiff",
  width = 12,
  height = 10,
  dpi = 300,
  compression = "lzw"
)

# ----------------------------------------------------------------------
------
# EXTRA STEP: GENE-SPECIFIC BOXPLOTS (Figure S2) - TIME-ONLY COLOR
# ----------------------------------------------------------------------
------
cat("\n=== GENERATING FIGURE S2: BOXPLOTS COLORED BY TIME ===\n")

library(ggtext)

# 1. HTML Labels for X-axis subscripts
pca_labels_html <- c(
  "Control" = "Control",
  "CO2"     = "CO<sub>2</sub>",
  "NaHCO3"  = "NaHCO<sub>3</sub>"
)

# 2. Target genes and labels
target_genes <- c("ACR0PX_RS03590", "ACR0PX_RS12845")
gene_labels <- c(
  "ACR0PX_RS03590" = "ACR0PX_RS03590\n(phosphoenolpyruvate carboxylase)",
  "ACR0PX_RS12845" = "ACR0PX_RS12845\n(carbonic anhydrase)"
)

# 3. Data Preparation
df_genes <- assay(vsd)[target_genes, , drop = FALSE] %>%
  as.data.frame() %>%
  rownames_to_column("locus_tag") %>%
  pivot_longer(-locus_tag, names_to = "sample", values_to = "expression")
%>%
  left_join(as.data.frame(colData) %>% rownames_to_column("sample"), by =
"sample") %>%
  mutate(
    gene_name = gene_labels[locus_tag],
    time = factor(time, levels = c("12", "18"))
  )
```

```
# 4. Create the Plot
fig_s2_time_color <- ggplot(df_genes, aes(x = condition, y = expression,
fill = time)) +
  # Grouped Boxplot (Whiskers included by default)
  geom_boxplot(
    position = position_dodge(width = 0.8),
    outlier.shape = NA,
    color = "black",
    alpha = 0.9
  ) +
  # Jittered biological replicates (matching the dodge)
  geom_point(
    position = position_jitterdodge(jitter.width = 0.1, dodge.width =
0.8),
    size = 1.2,
    color = "black",
    alpha = 0.7,
    show.legend = FALSE
  ) +
  facet_wrap(~gene_name, scales = "free_y") +
  # Apply the subscripts to the X-axis
  scale_x_discrete(labels = pca_labels_html) +
  # Distinct colors ONLY for time
  scale_fill_manual(
    values = c("12" = "#66c2a5", "18" = "#fc8d62"),
    labels = c("12 h", "18 h")
  ) +
  labs(
    x = "Condition",
    y = "VST Normalized Expression",
    fill = "Time"
  ) +
  publication_theme +
  theme(
    strip.text = element_text(size = 10, face = "bold.italic"),
    axis.text.x = element_markdown(size = 11), # Renders HTML tags
    legend.position = "right"
  )

# 5. Save as Figure_S2.tiff
output_s2_path <- file.path(output_dir, "Figure_S2.tiff")

agg_tiff(output_s2_path, width = 190, height = 105, units = "mm",
         res = 600, compression = "lzw")

print(fig_s2_time_color)

dev.off()

cat("âœ" Figure S2 (Time-colored Whiskers) saved to:", output_s2_path,
"\n")
```
