# Supplementary material for: Inorganic Carbon Modulates Emulsification Activity and Transcriptional Responses in Vreelandella zhaodongensis BS253
Source: Molecules. 2026 Jun 22;31(12):2182. doi: 10.3390/molecules31122182 (PMC13305022; doi:10.3390/molecules31122182)
Supplement: Supplementary file 1 [file molecules-31-02182-s001.zip › Supplementary Figure Captions.pdf]

Supplementary Figure Captions

Figure S1. Growth of strain BS253 under anaerobiosis and 5% CO2-enriched conditions: (A) Growth on saline agar plates incubated in an anaerobic jar system. (B) Growth on MSM medium supplemented with NaHCO3 under a 5% CO2-enriched atmosphere. (C) Growth on MSM medium without supplementation under a 5% CO2-enriched atmosphere.

Figure S2. Heatmap and hierarchical clustering of the top 100 most variable genes across experimental conditions and time points. Rows represent genes, and columns represent samples grouped by cultivation condition and sampling time. Colors indicate row-scaled expression values.

Figure S3. Expression of key carbon metabolism genes. Boxplots showing variance-stabilized normalized expression values for genes annotated as phosphoenolpyruvate carboxylase (ACR0PX_RS09580) and carbonic anhydrase (ACR0PX_RS13445) in Vreelandella zhaodongensis BS253 cultivated under Control, CO_2-enriched, and $NaHCO_3$-supplemented conditions at 12 h and 18 h. Colors indicate sampling time. Expression values are shown as VST-normalized counts obtained from RNA-seq data.

Figure S4. Transcriptional profiling of potential extracellular polysaccharide (EPS) biosynthetic genes in *V. zhaodongensis* BS253. The heatmap displays Z-score normalized expression levels of 115 genes associated with EPS assembly, export, and precursor metabolism across distinct cultivation conditions (Control, CO2, and NaHCO3) at 12 h and 18 h. Columns are grouped by experimental treatment and sampling time. The left annotation (Is DEG) indicates genes identified as significantly differentially expressed (|log2 Fold Change|>1, padj <0.05) in five key experimental contrasts: C 18v12 (Control 18 h vs. 12 h), CO2 18v12 (CO2 18 h vs. 12 h), Na 18v12 (NaHCO3 18 h vs. 12 h), Na v C (18) (NaHCO3 vs. Control at 18 h), and CO2 v C(18) (CO2 vs. Control at 18 h). The right annotation (Function) categorizes genes into four primary functional groups based on eggNOG-mapper results: Cell Wall/Envelope biogenesis (orange), Export/Polymerization (blue), Glycosyl-transferase activity (red), and Other EPS-related precursor metabolism (brown). High and low expression levels are represented by red and blue gradients, respectively.

Figure S5. Preliminary qualitative biochemical screening of the extracellular fraction. NC and PC indicate the negative control (water) and positive control (5% SDS), respectively: (A) Qualitative screening for neutral lipid-associated components using iodine/potassium iodide staining. (B) Qualitative screening for anionic functional groups using CTAB/methylene blue complexation. (C) Qualitative screening for amino-containing compounds using the ninhydrin assay.

(D) Qualitative screening for carbohydrate-associated components using the orcinol reaction.
