# Supplementary figures and images for: Inorganic Carbon Modulates Emulsification Activity and Transcriptional Responses in Vreelandella zhaodongensis BS253
Source: Molecules. 2026 Jun 22;31(12):2182. doi: 10.3390/molecules31122182 (PMC13305022; doi:10.3390/molecules31122182)

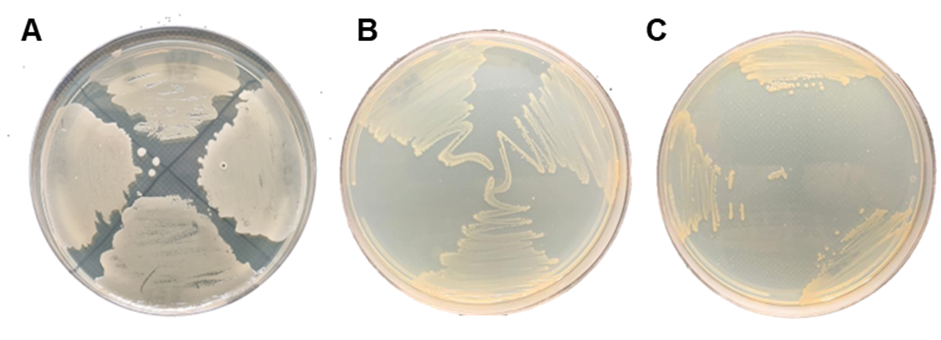

Supplement: Supplementary file 1 [file molecules-31-02182-s001.zip › Figure S1.tiff]

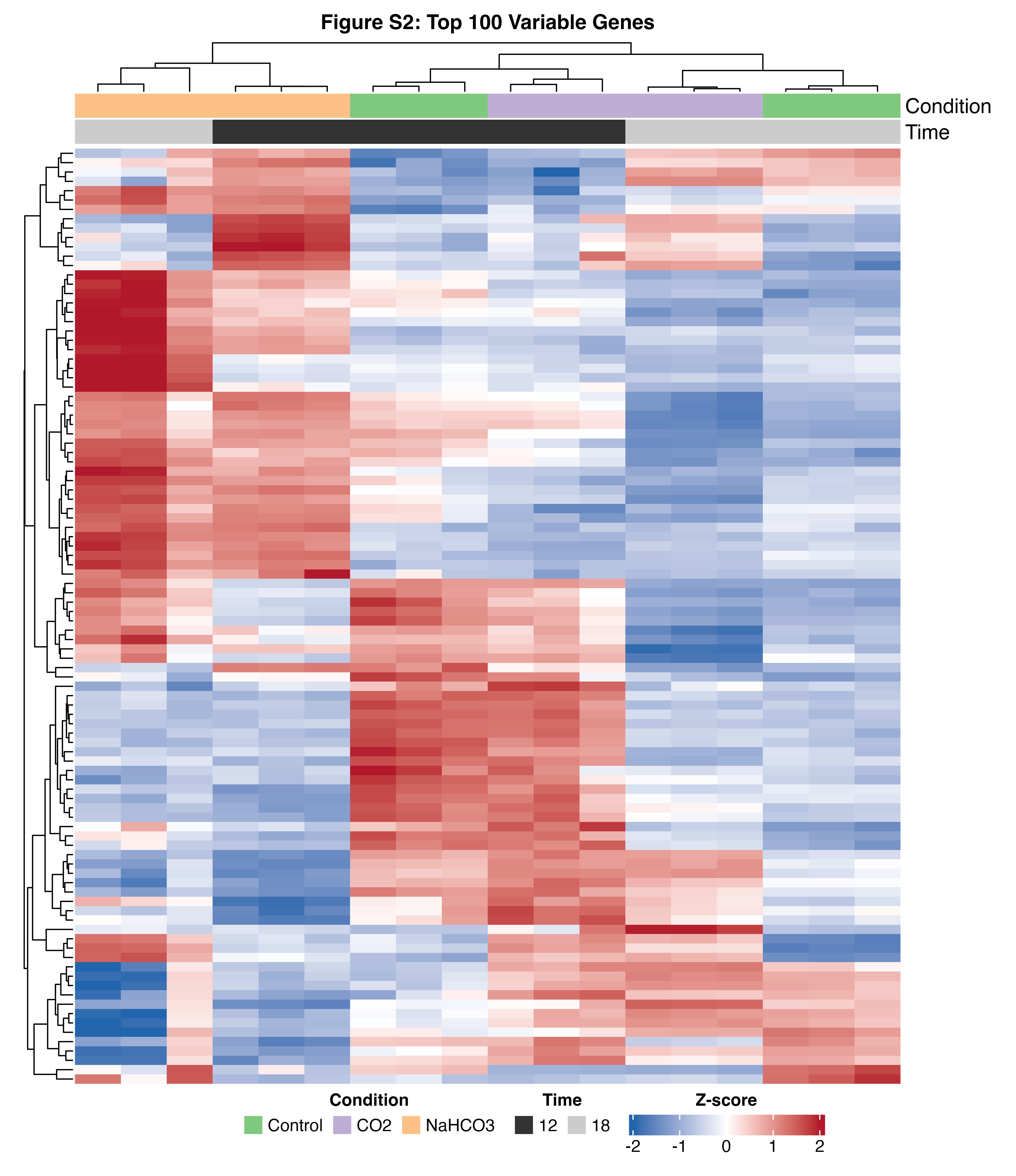

Supplement: Supplementary file 1 [file molecules-31-02182-s001.zip › Figure S2.tiff]

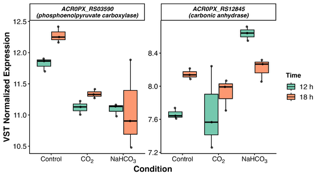

Supplement: Supplementary file 1 [file molecules-31-02182-s001.zip › Figure S3.tif]

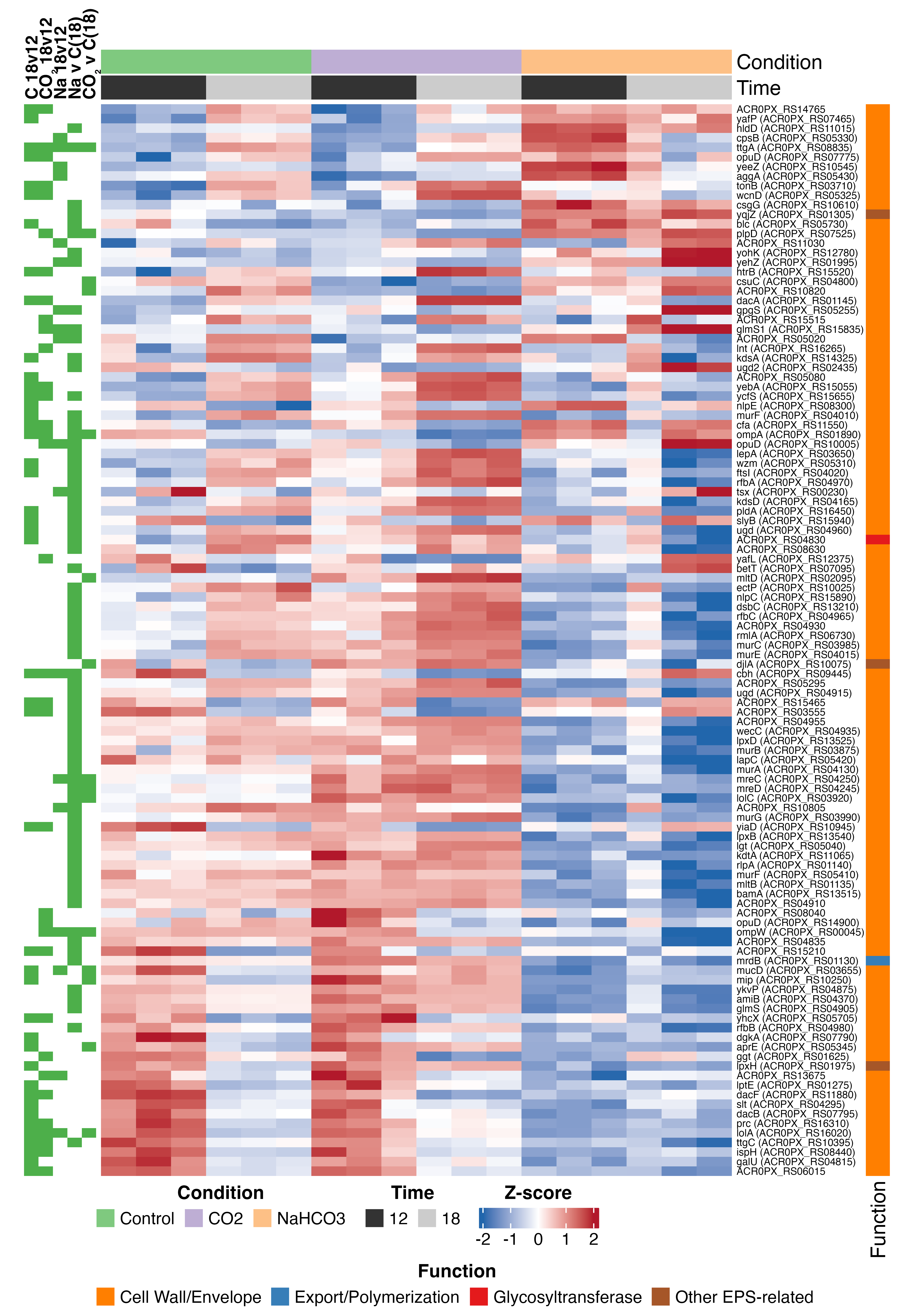

Supplement: Supplementary file 1 [file molecules-31-02182-s001.zip › Figure S4.tif]

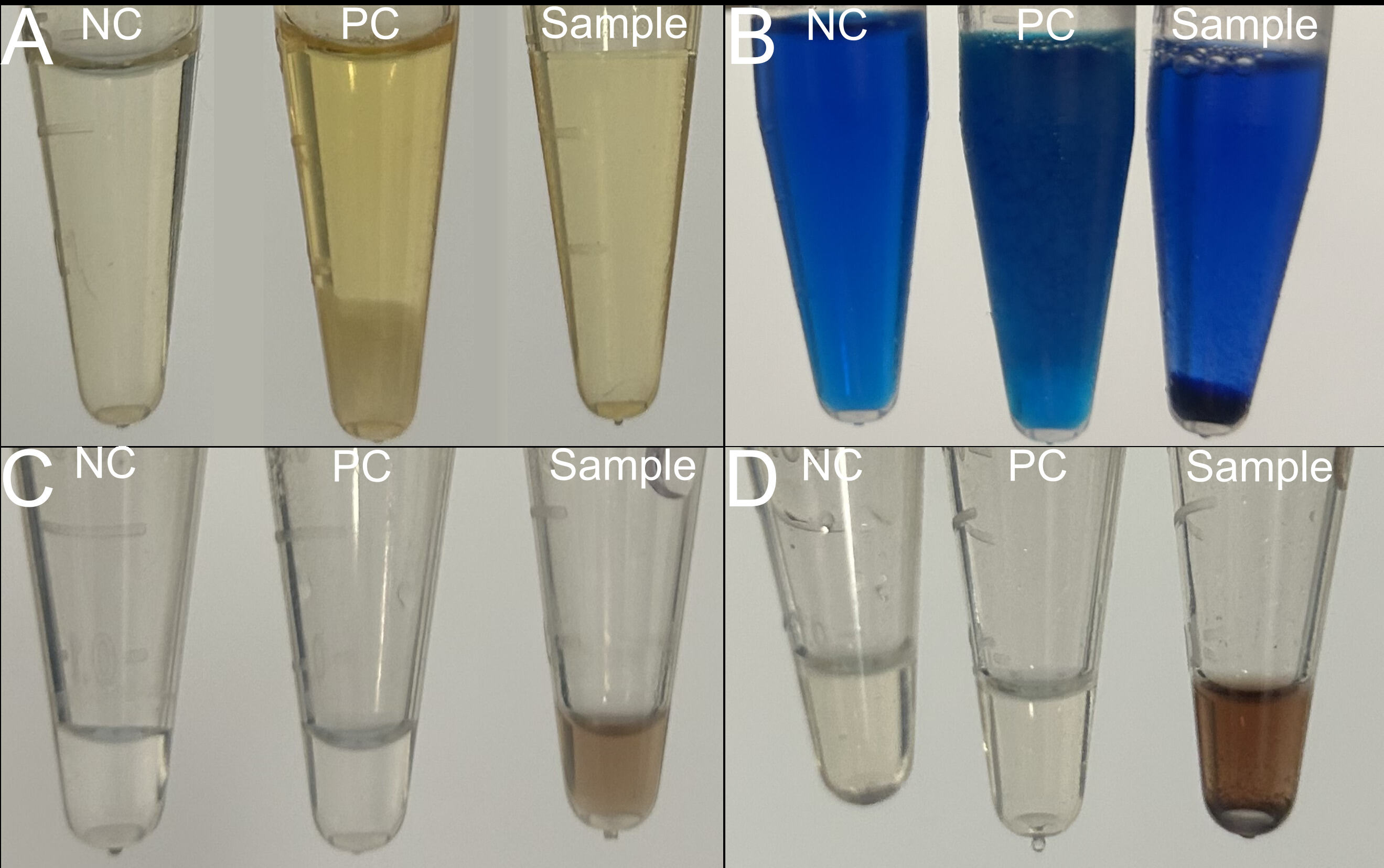

Supplement: Supplementary file 1 [file molecules-31-02182-s001.zip › Figure S5.tiff]
